# Dietary Fiber from Baijiu Distillers’ Grains Improves Glucose–Lipid Homeostasis via Gut–Liver Metabolic Remodeling

**DOI:** 10.3390/foods15122163

**Published:** 2026-06-15

**Authors:** Shangwu Chen, Kaizhang Wu, Wenqing Yu, Xiaoning Zhai, Zecheng Zhang, Yang Zheng, Jie Gao

**Affiliations:** 1 School of Light Industry and Food Engineering, Guangxi University, Nanning 530004, China; 2 Institute of Agricultural Product Processing, Chinese Academy of Agricultural Sciences, Beijing 100193, China

**Keywords:** dietary fiber, short-chain fatty acids, bile acid metabolism, hepatic metabolomics, PPAR-γ

## Abstract

Baijiu distillers’ grains (BDG), a major fermented cereal by-product of baijiu production, represent an underutilized source of structurally modified dietary fiber with potential value for functional food development. Here, we found that BDG-derived dietary fiber (BDG-DF), mainly composed of mannose (34.83 ± 0.38%) and xylose (35.14 ± 0.25%), promoted short-chain fatty acid production during in vitro fermentation, and its fermentation supernatants reduced IL-1β and TNF-α levels and modestly decreased IL-6 production in a Caco-2/HepG2 co-culture model. In T2D mice, BDG-DF improved glucose tolerance, with high-dose BDG-DF reducing the OGTT area under the curve by 12.4% compared with the T2D group, and alleviated hepatic steatosis. These effects were accompanied by enrichment of *Akkermansia* and *Bifidobacterium* and remodeling of bile acid profiles. High-dose BDG-DF was also associated with elevated CA and CDCA levels, altered TGR5/GLP-1 signaling, increased hepatic FXR expression, and reduced CYP7A1 expression. Integrated hepatic proteomics and metabolomics further indicated that BDG-DF was associated with changes in unsaturated fatty acid biosynthesis and PPAR-γ-related metabolic signaling. Overall, these findings suggest that BDG-DF may improve glucose–lipid homeostasis in association with gut microbiota and bile acid remodeling and hepatic PPAR-γ-related metabolic signaling.

## 1. Introduction

Dietary fiber (DF) from grains is a key dietary component that plays a well-recognized role in regulating glucose and lipid metabolism [1]. Emerging evidence establishes that cereal-derived dietary fiber can improve glucose tolerance, modulate lipid metabolism, and support metabolic health through multiple mechanisms, including delaying carbohydrate absorption and shaping gut microbiota composition [2]. As a result, grain-based dietary fiber has attracted considerable attention as a key nutritional factor in metabolic regulation [3]. The metabolic effects of dietary fiber are not determined solely by intake quantity, but are strongly influenced by its structural characteristics [4]. Features such as monosaccharide composition, branching patterns, and physical architecture govern how dietary fiber is accessed and fermented by gut microorganisms, thereby shaping microbial metabolism and the generation of downstream metabolites [5]. Consequently, structure-oriented studies have become increasingly important for understanding the functional diversity observed among different dietary fiber sources [6].

Most existing studies have focused on cereal-derived dietary fibers in their native or mildly processed forms [7]. In contrast, systematic investigations into fermentation-modified cereal dietary fibers remain relatively limited [8]. Previous research has demonstrated that fermentation can markedly reshape the physicochemical properties of dietary fiber, not only by altering molecular weight and branching structure, but also by modifying monosaccharide composition [9]. These structural changes can, in turn, influence fermentability and biological activity in the gut [10]. However, the specific structural features generated under different fermentation systems and raw material backgrounds, as well as their functional relevance for metabolic regulation, are still poorly understood [11]. Baijiu distillers’ grains (BDG) are a major by-product of baijiu production, generated through prolonged microbial saccharification and fermentation of cereal substrates such as sorghum, rice, wheat, and maize [12]. During this process, microbial enzymes and acidic fermentation conditions may partially remove easily degradable carbohydrates and modify the remaining insoluble fiber matrix, thereby changing its monosaccharide composition, surface morphology, and accessibility to gut microorganisms. Therefore, the long fermentation history of BDG may not merely represent a processing background, but may directly contribute to the formation of a structurally modified fiber fraction with distinct fermentability and metabolic effects. However, the specific structural features of BDG-derived dietary fiber (BDG-DF), its fermentability by gut microbiota, and its potential role in regulating glucose and lipid metabolism remain poorly understood. This knowledge gap has limited the value-added utilization of BDG-derived dietary fiber and its application in metabolic health-oriented functional foods.

Based on these considerations, we hypothesized that the fermentation-derived structural characteristics of BDG-DF may enhance its accessibility to gut microbiota, promote microbial metabolite production, and subsequently influence bile acid metabolism and hepatic metabolic signaling involved in glucose and lipid homeostasis. To test this hypothesis, we first characterized the structural and physicochemical properties of insoluble BDG-DF, including monosaccharide composition, surface morphology, Fourier-transform infrared spectra, and particle size distribution. We then evaluated its fermentability using an in vitro fecal fermentation system and assessed the biological effects of the fermentation supernatants in a Caco-2/HepG2 co-culture model. Furthermore, a high-fat diet combined with a streptozotocin-induced T2D mouse model was used to determine whether BDG-DF improves glucose tolerance, insulin sensitivity, serum lipid profiles, and hepatic lipid accumulation. To clarify the potential gut–liver metabolic links, we analyzed gut microbiota composition, short-chain fatty acids, bile acid profiles, and hepatic proteomic and metabolomic changes, with particular attention to bile acid-related receptor signaling and hepatic lipid metabolic pathways.

## 2. Materials and Methods

### 2.1. Reagents and Materials

Biochemical kits were sourced from Nanjing JianCheng Bioengineering Institute (Nanjing, China). Primary antibodies against *GAPDH*, *FXR*, *CYP7A1*, *CYP8A1*, and *PPAR-γ* were purchased from Proteintech Group (Wuhan, China). Animal whole protein extraction kits and total RNA extraction kits were procured from Sangon Biotech (Shanghai, China).

### 2.2. Extraction of Baijiu Distillers’ Grains–Dietary Fiber

Baijiu distillers’ grains (BDG) were obtained from the Chaozhou Branch of the Guangdong Laboratory of Chemistry and Fine Chemicals (Shantou, Guangdong Province, China). The BDG sample was collected from a rice-based baijiu fermentation/distillation process. In this process, rice served as the main brewing substrate, whereas rice husk was used as an auxiliary material to improve aeration and maintain the looseness of the solid fermentation matrix rather than as the principal fermentable raw material. After fermentation and distillation, the resulting BDG contained a substantial amount of insoluble plant residue, including rice-husk-derived fibrous components. Therefore, the recovered solid residue used in this study was enriched in insoluble fibrous material.

After washing, the BDG sample was passed through a 20-mesh sieve to remove debris, then milled. A 100 g portion was soaked in 500 mL of 1% (*w*/*v*) NaOH for 24 h, followed by centrifugation at 5000 rpm for 15 min to remove the alkali. This alkaline treatment was mainly used to remove alkali-soluble impurities and part of the hemicellulose fraction, thereby enriching the insoluble fiber fraction rather than fully preserving the native BDG matrix. The pellet was then resuspended in 500 mL of 3% (*w*/*v*) sodium chlorite, the pH was adjusted to 5.0 with lactic acid, and the suspension was bleached in an 85 °C water bath for 4 h. The sodium chlorite treatment under acidic conditions contributed to bleaching and partial delignification. Therefore, the obtained BDG-DF should be regarded as a chemically extracted, cellulose-rich insoluble dietary fiber fraction containing residual hemicellulose- and lignin-associated components. The resulting pale-yellow precipitate was collected by centrifugation, dried, milled, and passed through a 100-mesh sieve to obtain BDG-derived dietary fiber (BDG-DF). The basic composition of BDG-DF is shown in Supplementary Table S1.

### 2.3. Compositional, Morphological, and Physicochemical Characterization of BDG-DF

The compositional, morphological, and physicochemical properties of BDG-DF were analyzed, including monosaccharide composition, Fourier-transform infrared spectra, X-ray diffraction patterns, particle size distribution, Scanning electron microscopy, and rheological behavior. Detailed procedures for sample preparation and instrument settings are provided in Section S1 of the Supplementary Data.

### 2.4. In Vitro Fecal Fermentation

In vitro colonic fermentation was performed according to a previously reported method with minor modifications to evaluate the fermentability of digested BDG-DF [13]. Fresh fecal samples were collected from four healthy adult donors aged 20–25 years, including two males and two females. None of the donors had taken antibiotics, probiotics, prebiotics, or other medications known to affect gut microbiota within the previous three months, and none had a history of gastrointestinal disorders. Equal amounts of feces from the four donors were pooled to reduce inter-individual variability and to obtain a representative mixed fecal inoculum for substrate fermentation. All fecal samples were transferred into an anaerobic workstation immediately after collection and processed under anaerobic conditions. The anaerobic atmosphere consisted of 85% N_2_, 10% CO_2_, and 5% H_2_. The pooled fecal sample was homogenized in sterile phosphate-buffered saline (PBS, pH 7.0) to prepare a 32% (*w*/*v*) fecal slurry. The slurry was centrifuged at 500 rpm for 5 min at 4 °C, and the supernatant was collected as the fecal inoculum.

Anaerobic fermentation was carried out in basal medium containing peptone, yeast extract, mineral salts, hemin, L-cysteine, bile salts, Tween 80, and resazurin. Before inoculation, the medium was pre-reduced and equilibrated under anaerobic conditions, and the absence of oxygen was monitored by the colorless state of resazurin. Four experimental groups were established: Blank, Inulin, DF-L, and DF-H. The Blank group contained basal medium and fecal inoculum without added substrate; the Inulin group contained basal medium, fecal inoculum, and inulin as a positive fermentable substrate control; and the DF-L and DF-H groups contained basal medium, fecal inoculum, and 2.5% or 7.5% digested BDG-DF, respectively. Each fermentation vessel received 2.0 mL of fecal inoculum, and the final volume was adjusted with basal medium to the same volume across all groups. Fermentation was conducted at 37 °C for 24 h under anaerobic conditions. Samples were collected at 0, 3, 5, 8, 12, and 24 h, immediately cooled on ice, and centrifuged. The fermentation supernatants were collected for SCFA analysis. For cell experiments, the 24 h fermentation supernatants were further centrifuged and filtered through a 0.22 μm membrane to remove residual microorganisms and particles before being added to the cell culture medium. All fermentations were performed in triplicate.

### 2.5. Cell Culture and Caco-2/HepG2 Co-Culture Experiment

The Caco-2/HepG2 co-culture model was established based on previously reported methods with minor modifications [14]. HepG2 and Caco-2 cells were cultured in high-glucose Dulbecco’s modified Eagle’s medium (DMEM, Nanjing JianCheng Bioengineering Institute, Nanjng, China) supplemented with 10% heat-inactivated fetal bovine serum and 1% penicillin–streptomycin. Cells were maintained at 37 °C in a humidified incubator containing 5% CO_2_. Cells in the logarithmic growth phase were harvested by trypsinization, resuspended in complete medium, and seeded into sterile culture plates for subsequent experiments. HepG2-alone assays were first performed to evaluate the cytotoxicity of BDG-DF fermentation supernatants and to determine suitable intervention concentrations. Briefly, HepG2 cells were seeded into 96-well plates and allowed to attach for 24 h. The cells were then treated with sterile-filtered BDG-DF fermentation supernatants at 0, 0.5%, 1%, 2%, 5%, and 10% (*v*/*v*) for 24 h. Cell viability was determined using the CCK-8 assay according to the manufacturer’s instructions. Based on the cell viability results, 0.5% and 1.0% fermentation supernatants were selected for subsequent intervention experiments because these concentrations maintained cell viability while allowing evaluation of biological activity.

To further evaluate the biological effects of BDG-DF fermentation-derived metabolites in a more physiologically relevant gut–liver interface model, a Caco-2/HepG2 Transwell co-culture system was established. The HepG2-alone assay was used for concentration screening, whereas the Caco-2/HepG2 co-culture model was used to mimic intestinal exposure to fermentation-derived metabolites and downstream hepatic responses. Caco-2 cells (2.5 × 10^5^ cells/mL) were seeded in the apical chamber of Transwell inserts to form a confluent epithelial-like monolayer, while HepG2 cells (1 × 10^5^ cells/mL) were seeded in the basolateral chamber. Oleic acid treatment was applied to induce lipid metabolic stress in HepG2 cells based on established HepG2 lipid accumulation models. After cell attachment and stabilization, oleic acid (1 μg/mL) was added to the basolateral chamber for 4 h to induce lipid metabolic stress in HepG2 cells. The cells were then divided into four groups: normal control group (NC), model group (MD), 0.5% BDG-DF fermentation supernatant group, and 1.0% BDG-DF fermentation supernatant group. In the intervention groups, sterile-filtered BDG-DF fermentation supernatants were added to the apical chamber to mimic intestinal exposure to fermentation-derived metabolites. After 24 h of incubation, culture supernatants from the basolateral chamber were collected and centrifuged to remove cell debris. The resulting supernatants were used for the determination of cytokine levels, including IL-1β, TNF-α, IL-6, and IL-10. The Caco-2/HepG2 co-culture model in this study was designed to evaluate the effects of fermentation-derived metabolites on hepatic inflammatory responses after intestinal epithelial exposure. It was not intended as a direct assay of intestinal barrier integrity. Therefore, transepithelial electrical resistance and paracellular permeability were not measured.

### 2.6. Animals and Experimental Design

All animal procedures were approved by the Institutional Animal Care and Use Committee of Guangxi University (GXU-2024-0035). Male C57BL/6J mice, weighing 18–22 g, were purchased from SPF Beijing Biotechnology Co., Ltd. (Beijing, China). All mice were housed under controlled conditions of 22 ± 3 °C, 50 ± 5% relative humidity, and a 12 h light/dark cycle, with ad libitum access to food and water. After 1 week of acclimation, mice were randomly assigned to either a normal chow group or a high-fat diet (HFD) group. Thirty-two mice received HFD for 8 weeks to induce obesity and insulin resistance, while the remaining mice were maintained on standard chow. In week 9, obesity-prone mice were rendered diabetic with streptozotocin (STZ). A freshly prepared 1% (*w*/*v*) STZ solution in sodium citrate buffer (pH 4.5; 10 mg/mL) was kept on ice and protected from light. Following a 12 h fast with free access to water, mice were injected intraperitoneally with STZ at 120 mg/kg. Food was returned 2 h after injection; 5% glucose water was provided at 4 h and replaced with regular water at 10 h. Vehicle control mice received citrate buffer only. One week later, fasting blood glucose > 11.1 mmol/L was taken as evidence of diabetes. The HFD/STZ model was used because it combines diet-induced insulin resistance with STZ-induced pancreatic β-cell injury, thereby reproducing key features of type 2 diabetes, including hyperglycemia, impaired glucose tolerance, insulin resistance, and lipid metabolic disturbance. However, because STZ has direct pancreatotoxic effects, this model may include a substantial β-cell injury component and should not be interpreted as a purely diet-induced insulin resistance model.

As shown in one of the Figures, mice were assigned to five groups (*n* = 8/group): NC, normal chow control group; T2D, HFD/STZ-induced diabetic model group; Metformin, HFD/STZ-induced diabetic mice treated with metformin at 100 mg/kg by gavage; DF-L, HFD/STZ-induced diabetic mice fed a diet supplemented with 2.5% BDG-DF; and DF-H, HFD/STZ-induced diabetic mice fed a diet supplemented with 7.5% BDG-DF. The sample size of eight mice per group was selected based on previous comparable HFD/STZ-induced T2D mouse studies and dietary fiber intervention experiments, while also considering ethical reduction of animal use. This sample size was considered sufficient to detect treatment-related changes in major metabolic endpoints, including fasting blood glucose, OGTT, serum lipid profiles, and hepatic lipid accumulation. The experimental timeline was summarized in one of the Figures. Briefly, the study included 1 week of acclimation, 8 weeks of HFD feeding, STZ injection in week 9, diabetes confirmation 1 week later, 6 weeks of dietary intervention, and final sample collection. At the end of the intervention, mice were euthanized; liver, kidney, colon, and epididymal white adipose tissue were excised, rinsed in saline, blotted, and weighed. Blood, liver, colon, and colonic contents were snap-frozen in liquid nitrogen and stored at −80 °C until analysis.

### 2.7. Fasting Blood Glucose and Oral Glucose Tolerance Test (OGTT)

Fasting blood glucose was measured from the tail vein using a glucometer. For OGTT, mice were fasted for 10–12 h with free access to water. Baseline blood glucose was recorded at 0 min, followed by oral gavage of D-glucose at 2 g/kg body weight prepared as a 20% (*w*/*v*) solution (200 mg/mL). Blood glucose was measured at 15, 30, 60, 90, and 120 min post gavage.

### 2.8. Biochemical Analysis

Serum TC, TG, LDL-C, HDL-C, ALT, and AST were determined with commercial assay kits according to the manufacturers’ protocols. Liver tissues were homogenized in ice-cold saline at a 1:9 ratio, followed by centrifugation at 3000 rpm for 10 min. The supernatants were collected for protein normalization and subsequent measurement of hepatic TC, TG, and glycogen. Serum insulin was measured using an ELISA kit.

### 2.9. Histopathological Analysis

Liver and colon tissues were fixed in 4% paraformaldehyde and embedded in paraffin. Serial sections (4 µm) were prepared for hematoxylin–eosin (H&E) staining; Oil Red O staining was performed to visualize neutral lipid deposition. Stained sections were imaged by light microscopy for histopathological assessment.

### 2.10. 16S rRNA Gene and Bioinformatics Analysis

Intestinal microbiome analysis was performed according to a previously described method with minor modifications [14]. Briefly, the V3–V4 hypervariable regions of the bacterial 16S rRNA gene were amplified using universal primers 338F (5′-ACTCCTACGGGAGGCAGCAG-3′) and 806R (5′-GGACTACHVGGGTWTCTAAT-3′). Amplicons were sequenced on an Illumina paired-end platform. Raw reads were processed using the QIIME2 pipeline, including demultiplexing, quality trimming, denoising, merging, and chimera removal with the DADA2 plugin. High-quality reads were resolved into amplicon sequence variants (ASVs). Taxonomic annotation of ASVs was performed using the QIIME2 feature-classifier against the Greengenes 13_8 database clustered at 99% sequence similarity. The classifier was trained for the V3–V4 region amplified by the 338F/806R primer pair. Greengenes 13_8 was selected because it has been widely used in mouse gut microbiota studies and is compatible with our previous analytical pipeline, allowing comparison with earlier datasets generated in our laboratory. However, because Greengenes is a legacy 16S reference database, taxonomic interpretations were mainly made at the genus level, and species-level assignments were avoided unless strongly supported. Microbial community profiles were then used for downstream diversity, differential abundance, and correlation analyses.

### 2.11. Untargeted Metabolome Analysis

Metabolites from fecal and liver samples were extracted according to established protocols with slight adjustments [15]. Untargeted metabolomics was performed on an HPLC–MS/MS system equipped with a SCIEX X500R QTOF (Marlborough, MA, USA) mass spectrometer. Samples were separated using methanol and 0.1% formic acid as mobile phases, with an injection volume of 1 μL, a flow rate of 0.2 mL/min, and a column temperature of 30 °C. Data were acquired in both positive and negative ESI modes over an *m*/*z* range of 100–1000. The raw metabolomic data were processed and statistically evaluated using MetaboAnalyst 6.0.

### 2.12. Targeted Bile Acid Quantification

Targeted bile acids were quantified following a published method [16]. Sample preparation and LC-MS analysis were performed as described in Section 2.7. Standards were prepared at 10, 20, 30, 40, 60, 100, 500, 1000, 2000, 5000, and 10,000 ng/mL to generate calibration curves.

### 2.13. Determination of SCFAs

Fecal SCFAs were quantified by GC–MS. Fresh fecal pellets collected at the end of the intervention were snap-frozen in liquid nitrogen and stored at −80 °C. Approximately 5.0 mg of feces was homogenized with formic-acidified water and centrifuged at 12,000× *g* for 15 min at 4 °C. The supernatant was extracted with ethyl acetate containing 4-methylvaleric acid as the internal standard, filtered through a 0.22 μm membrane, and analyzed on an Agilent 7890B GC–MS system with a 5977A detector (Santa Clara, CA, USA). SCFAs were identified and quantified using authentic standards.

### 2.14. Liver Proteomics Sample Preparation and LC–MS/MS Analysis

#### 2.14.1. Protein Extraction and QC

Frozen liver was homogenized on ice in SDS lysis buffer supplemented with protease inhibitor and PMSF (1 mM). Lysates were sonicated on ice (≈2 min) and cleared by centrifugation (10,000 rpm, 4 °C, 10 min). Supernatants were collected as total protein and quantified by BCA. Aliquots were assessed by SDS-PAGE (10% gel; Coomassie staining) to verify integrity.

#### 2.14.2. Reduction, Alkylation, and Digestion

Per sample, 0.5 mg protein was reduced with DTT (5 mM, 55 °C, 30 min) and alkylated with iodoacetamide (10 mM, room temperature, dark, 15 min). Proteins were precipitated with six volumes of cold acetone (−20 °C, overnight), pelleted (5000 rpm, 4 °C, 10 min), and air-dried. Pellets were resuspended in 25 mM ammonium bicarbonate and digested with trypsin (Trypsin-TPCK, Sangon Biotech, Shnaghai, China) at 1:50 (*w*/*w*) for 12 h at 37 °C, followed by a second addition at 1:100 (*w*/*w*) for 6 h. Peptide yield was determined by BCA; 400 µg was taken forward.

#### 2.14.3. Peptide Cleanup

Samples were acidified (final 1% TFA), clarified (2000 rpm, 4 °C, 15 min), and desalted on C18 cartridges (Waters, Milford, MA, USA). Columns were conditioned with methanol and equilibration buffers, samples loaded, washed (0.2% TFA in water), and eluted with 90% acetonitrile/0.2% TFA. Eluates were dried or stored at −80 °C until LC-MS/MS.

#### 2.14.4. LC-MS/MS Acquisition

Peptides, 1 μg per sample, were separated on a Prominence Plus LC-30A system equipped with a Shim-pack GIS C18 (Shimadzu, Kyoto, Japan) column and analyzed using a SCIEX X500R QTOF (Marlborough, MA, USA) mass spectrometer. A 90 min gradient was applied with water containing 0.1% formic acid as mobile phase A and acetonitrile containing 0.1% formic acid as mobile phase B. Data were acquired in positive IDA-TOF MS/MS mode over an MS1 range of 350–1500 *m*/*z*, with rolling collision energy used for MS/MS fragmentation. Proteomic raw files were processed using DIA-NN, followed by enrichment analysis with Metascape, v3.5.2.

### 2.15. Real-Time Quantitative PCR

Colon and liver tissues (50–100 mg) were placed in RNase-free tubes, and total RNA was extracted with TRIzol (Molecular Research Center, Inc., Cincinnati, OH, USA) following the manufacturer’s instructions. RNA purity was assessed spectrophotometrically; samples with an A260/A280 ratio of 1.8–2.2 were used for downstream assays. First-strand cDNA was synthesized according to the reverse-transcription kit protocol and stored at −80 °C. Primer sequences are listed in Table S2.

### 2.16. Western Blot Analysis

Liver or colon tissues were lysed in SDS lysis buffer, and protein concentrations were measured using a BCA assay. Equal amounts of protein were mixed with loading buffer, denatured at 95 °C, separated by 10% SDS-PAGE, and transferred onto PVDF membranes. After blocking with 5% skim milk, the membranes were incubated overnight at 4 °C with primary antibodies, followed by HRP-conjugated secondary antibodies. Protein bands were visualized using an ECL detection system.

### 2.17. Statistical Analysis

All experiments were performed in triplicate or with additional biological replicates when required. Data are presented as mean ± standard deviation (SD). For biochemical, histological, qPCR, Western blot, SCFA, and targeted bile acid data, group differences were evaluated by one-way analysis of variance (ANOVA), followed by Tukey’s post hoc test for multiple comparisons. Statistical significance was defined as *p* < 0.05.

For microbiome, metabolomic, and proteomic datasets, multiple-testing correction was performed using the Benjamini–Hochberg false discovery rate (FDR) method. Differential metabolites and proteins were identified using combined criteria of fold change and FDR-adjusted *p*-value. For fecal metabolomic analysis, metabolites with fold change > 2 or <0.5 and FDR-adjusted *p* < 0.05 were considered significantly altered. For hepatic metabolomic analysis, metabolites with fold change > 1.2 or <0.83 and FDR-adjusted *p* < 0.05 were selected for downstream analysis. For proteomic analysis, differentially expressed proteins were defined using fold change > 2 or <0.5 and FDR-adjusted *p* < 0.05. For pathway enrichment analysis, enrichment *p*-values were also corrected by the Benjamini–Hochberg method, and pathways with adjusted *p* < 0.05 were considered significantly enriched.

Multivariate analyses were performed after data normalization, log transformation, and scaling where appropriate. Principal component analysis (PCA) was used as an unsupervised method to evaluate global sample distribution. Partial least-squares discriminant analysis (PLS-DA) was used as a supervised method to visualize group separation. To assess model robustness and reduce the risk of overfitting, PLS-DA models were evaluated by cross-validation and permutation testing. Model quality was assessed using R^2^ and Q^2^ values, and permutation tests were performed to confirm that the original model outperformed randomly permuted class labels. PLS-DA results were interpreted together with PCA, univariate statistics, FDR correction, and pathway-level evidence rather than used alone for biomarker selection.

## 3. Results

### 3.1. Structural and Physicochemical Properties of BDG-DF

**Figure 1:**
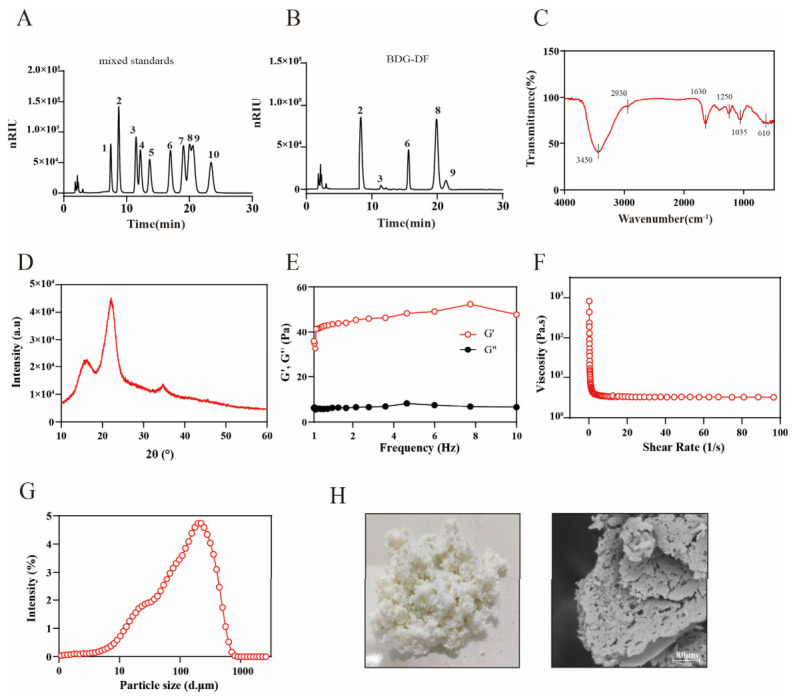
Compositional and physicochemical characteristics of BDG-DF. (**A**,**B**) Monosaccharide composition analysis. (**C**) Fourier transform infrared spectroscopy. (**D**) The X-ray diffraction spectra. (**E**) Dynamic rheological curves. (**F**) Apparent viscosity of BDG-DF as a function of shear rate. (**G**) The diameter distribution. (**H**) Macroscopic appearance and scanning electron microscopy (×800). The number 1–10 represent mannose acid, mannose, Ribose, glucose acid, galacturonic acid, glucose, galactose, xylose, arabinose, and fucose, respectively. Values are expressed as means ± SD (*n* = 3 for each group).

The insoluble dietary fiber isolated from baijiu distillers’ grains (BDG-DF) was mainly composed of cellulose (57.07 ± 1.03%), hemicellulose (12.26 ± 0.72%) and lignin (6.14 ± 0.29%) (Table S1). The structural and physicochemical properties of BDG-DF are shown in Figure 1. Monosaccharide analysis revealed that BDG-DF was mainly composed of glucose (28.35 ± 0.12%), mannose (34.83 ± 0.38%), and xylose (35.14 ± 0.25%) (Figure 1A,B). The FT-IR spectrum of BDG-DF exhibited a broad absorption band around 3450 cm^−1^, which was attributed to O-H stretching vibrations, and a peak near 2930 cm^−1^ corresponding to C–H stretching vibrations (Figure 1C). Additional absorption signals at approximately 1630, 1250, 1035, and 610 cm^−1^ suggested the presence of characteristic polysaccharide functional groups and glycosidic bond-related vibrations.

The X-ray diffraction pattern showed a major broad diffraction peak centered around 2θ = 20–25°, indicating that BDG-DF possessed a partially ordered crystalline structure (Figure 1D). Dynamic rheological analysis demonstrated that the storage modulus (G′) was consistently higher than the loss modulus (G″) over the tested frequency range (Figure 1E), suggesting that BDG-DF exhibited weak gel-like viscoelastic behavior. In addition, the apparent viscosity of BDG-DF decreased markedly with increasing shear rate (Figure 1F), indicating a typical shear-thinning behavior. Particle size analysis showed that BDG-DF displayed a broad size distribution, with the main particle population concentrated in the range of approximately 50–300 μm (Figure 1G). Macroscopic observation and scanning electron microscopy further revealed that BDG-DF presented a loose, porous, and irregular aggregated structure with a rough surface morphology (Figure 1H). These results collectively indicate that BDG-DF possesses distinct compositional, structural, and physicochemical characteristics. These features may influence its fermentability, although their direct contribution to biological activity requires further functional validation.

### 3.2. In Vitro Fermentability of BDG-DF and Biological Effects of Its Fermentation Supernatants

**Figure 2:**
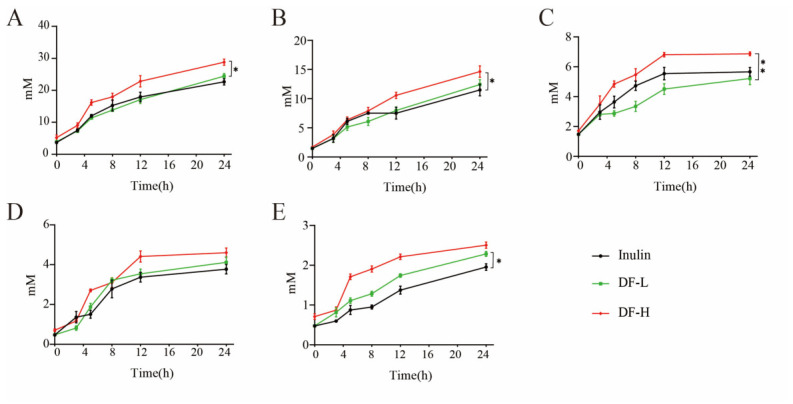

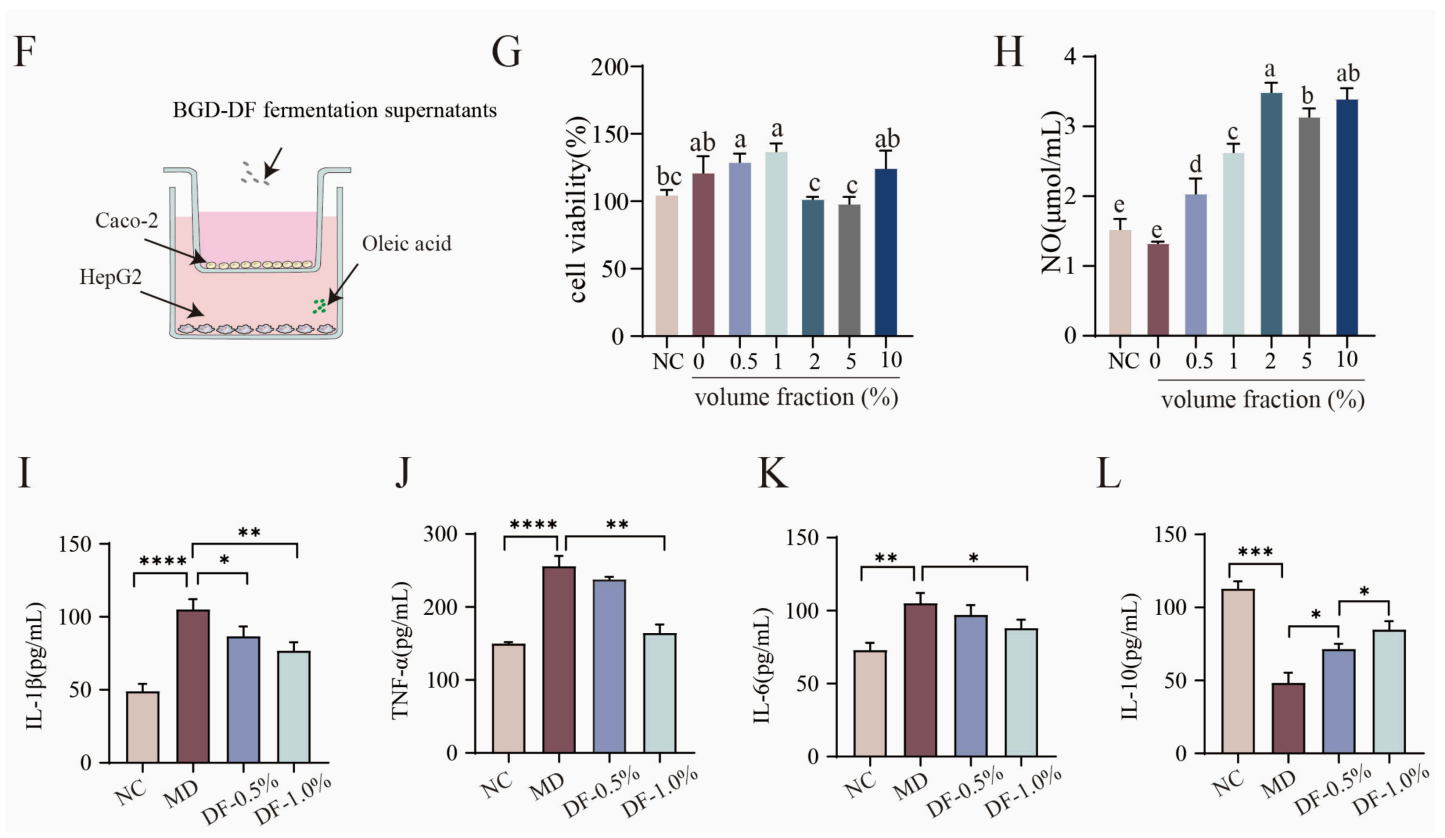
In vitro fermentability and cellular effects of BDG-DF fermentation supernatants. The changes of Acetate (**A**), Propionate (**B**), Butyrate (**C**), Isovalerate (**D**), and Total SCFAs (**E**) contents during the whole fermentation process of Inulin, DF-L, and DF-H. (**F**) Schematic representation of the Caco-2/HepG2 co-culture model. Effect of BDG-DF fermentation broths on the propagation (**G**) and NO release (**H**) of HepG2 cell. (**I**–**L**) Effects of BDG-DF treatment on IL-1β, TNF-α, IL-6, and IL-10 levels in Caco-2/HepG2 cells. Results are expressed as means ± SD (*n* = 3). Different letters indicate a significant difference between groups, *p* < 0.05, * *p* < 0.05, ** *p* < 0.01, *** *p* < 0.001, **** *p* < 0.0001.

As shown in Figure 2A–E, acetate, propionate, butyrate, isovalerate, and total SCFAs increased progressively during the 24 h fermentation. Both DF-L and DF-H exhibited higher SCFA production compared with inulin, particularly at the later fermentation stage (12–24 h). Among them, DF-H consistently showed the highest levels of SCFAs. The effects of BDG-DF fermentation supernatants on HepG2 cell viability are presented in Figure 2G. Treatment with low volume fractions (0.5–1%) slightly increased or maintained cell viability compared with the control group, whereas higher concentrations (≥2%) reduced cell viability. Based on these results, 0.5% and 1.0% were selected for subsequent experiments. As shown in Figure 2H, BDG-DF fermentation supernatants significantly increased NO production in HepG2 cells in a dose-dependent manner. Compared with the negative control, NO levels were markedly elevated at concentrations of 1–10% (*p* < 0.05), suggesting that fermentation-derived metabolites may enhance cellular signaling or redox activity. The co-culture model is illustrated in Figure 2F. As shown in Figure 2I–L, oleic acid stimulation significantly increased the levels of pro-inflammatory cytokines (IL-1β, TNF-α, and IL-6) compared with the normal control group. Treatment with BDG-DF fermentation supernatants (0.5% and 1.0%) significantly reduced the levels of IL-1β and TNF-α (*p* < 0.05), and slightly decreased IL-6 levels. Meanwhile, the anti-inflammatory cytokine IL-10 was significantly increased after BDG-DF treatment. Therefore, the increased SCFA production observed during in vitro fermentation provides a metabolic rationale for the subsequent in vivo findings, including improved glucose tolerance, colon integrity, lipid metabolism, and gut–liver metabolic remodeling. These data support the hypothesis that BDG-DF may act, at least in part, through microbiota-derived metabolites, although the causal contribution of SCFAs requires further validation.

### 3.3. Glycemic Control and Colon Integrity in T2D Mice Treated with BDG-DF

**Figure 3:**
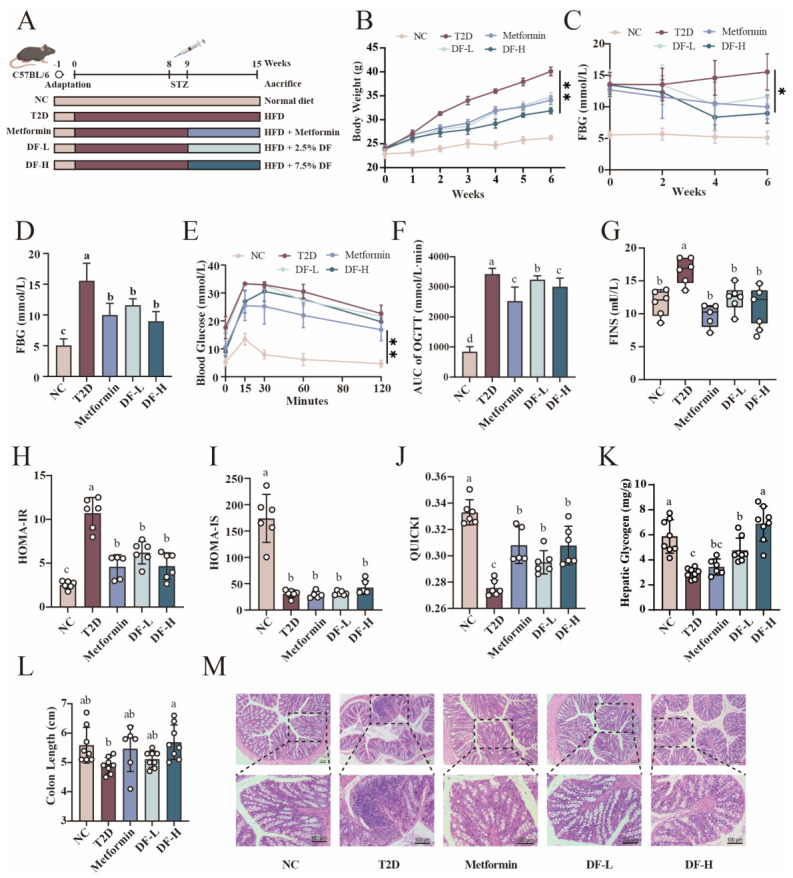
Glycemic control and colon integrity in T2D mice treated with BDG-DF. (**A**) Schematic of the animal experiment design. Experimental timeline of the HFD/STZ-induced T2D mouse model and BDG-DF intervention. After 1 week of acclimation, mice were fed either normal chow or HFD for 8 weeks. HFD-fed mice received STZ injection at week 9, and diabetes was confirmed 1 week later based on fasting blood glucose > 11.1 mmol/L. Mice then received metformin, low-dose BDG-DF, or high-dose BDG-DF intervention for 6 weeks before sample collection. (**B**) Body weight change. (**C**) FBG. (**D**) FBG at the end of intervention. (**E**) Blood glucose curve of OGTT. (**F**) AUC of OGTT. (**G**) FINS. (**H**) HOMA-IR. (**I**) HOMA-IS. (**J**) QUICKI. (**K**) Hepatic glycogen. (**L**) Colon length. (**M**) Histological colon tissue stained with H&E. Values are expressed as means ± SD (*n* = 8 for each group). Different letters indicate a significant difference between groups, * *p* < 0.05, ** *p* < 0.01.

The experimental design is illustrated in Figure 3A. After induction of type 2 diabetes, T2D mice exhibited a progressive decline in body weight compared with the NC group, whereas metformin and BDG-DF intervention partially attenuated diabetes-associated weight loss (Figure 3B). At the end of the intervention, body weight in the Metformin and DF-H groups was significantly higher than that in the T2D group, indicating an improved metabolic status. Fasting blood glucose in NC remained low and stable throughout. DF-L and DF-H followed comparable trajectories, falling below T2D from week 2 onward and, despite a slight rise in the final week, remaining significantly lower than T2D (*p* < 0.05; Figure 3C,D). During the oral glucose tolerance test (OGTT), blood glucose levels in the DF-L group began to decline after 30 min, with a modestly faster reduction rate than that observed in the T2D group (Figure 3E). Notably, glucose excursions in the Metformin and DF-H groups were consistently lower than those in the T2D group, with the area under the curve (AUC) reduced by 26.3% and 12.4%, respectively (Figure 3F).

Fasting insulin was elevated in T2D versus NC (*p* < 0.05; Figure 3G) and was significantly reduced by all interventions. Concordantly, T2D exhibited higher HOMA-IR and lower HOMA-IS than NC, whereas all three treatments lowered insulin (*p* < 0.05; Figure 3H,I) and increased QUICKI relative to T2D (*p* < 0.05; Figure 3J). Hepatic glycogen was markedly depleted in T2D but was restored by DF in a dose-dependent manner, with DF-H approaching NC and surpassing DF-L (Figure 3K). Compared with the T2D group, all intervention groups partially reversed diabetes-induced colon shortening. Among them, the DF-H group exhibited the most pronounced recovery, with colon length not significantly different from that of the NC group and significantly greater than that observed in the Metformin and DF-L groups (*p* < 0.05; Figure 3L). Histopathological analysis and macroscopic observations further demonstrated that DF intervention, particularly at a high dose, effectively alleviated colonic mucosal damage, improved intestinal gland architecture, and reversed colon shortening (Figure 3M).

### 3.4. Serum Lipid Profiles and Hepatic Steatosis in T2D Mice Treated with BDG-DF

**Figure 4:**
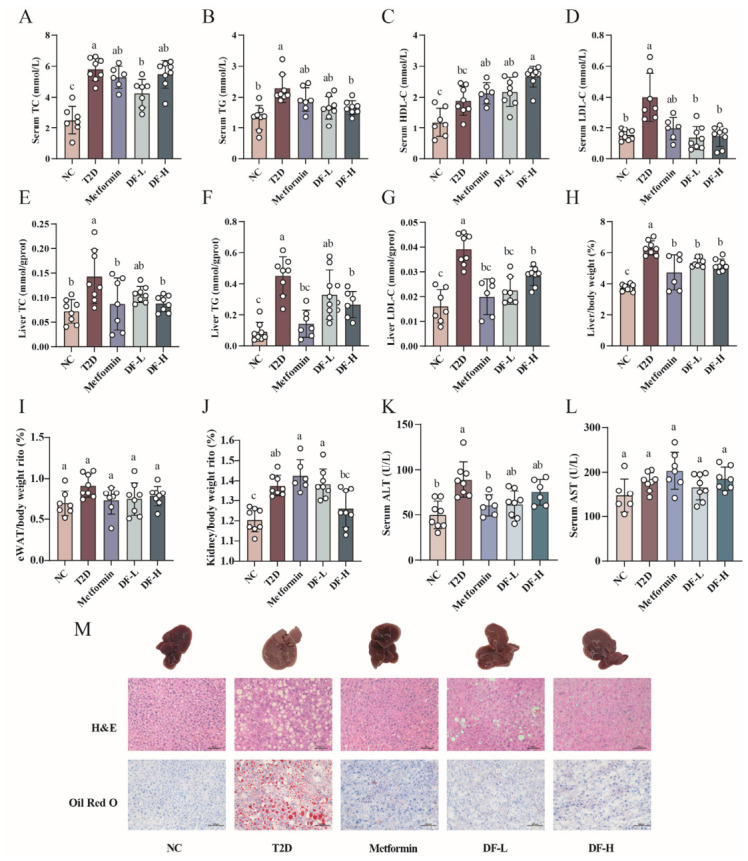
Serum lipid profiles and hepatic steatosis in T2D mice treated with BDG-DF. Serum lipid levels: (**A**) TC. (**B**) TG. (**C**) HDL-C. (**D**) LDL-C. Liver lipid levels in mice: (**E**) TC. (**F**) TG. (**G**) LDL-C. (**H**) Liver-to-body weight ratio. (**I**) Epididymal white adipose tissue ratio. (**J**) Kidney-to-body weight ratio. (**K**) ALT. (**L**) AST. (**M**) H&E staining and Oil red O staining of liver tissue. Values are expressed as means ± SD (*n* = 8 for each group). Different letters indicate a significant difference between groups, *p* < 0.05.

As shown in Figure 4A–D, the T2D group exhibited significant elevations in serum total cholesterol (TC), triglycerides (TG), and LDL-cholesterol (LDL-C). DF-L reduced TC and LDL-C, whereas DF-H significantly lowered TG and LDL-C and concurrently increased HDL-C (*p* < 0.05). To assess hepatic lipid deposition after distillers’ grain fiber intervention, hepatic lipids were quantified using commercial kits. In DF-L, hepatic TC, TG, and LDL-C trended lower than T2D without reaching significance (Figure 4E–G). The liver index (liver/body mass) was significantly reduced in Metformin, DF-L, and DF-H versus T2D (*p* < 0.05; Figure 4H). Relative epididymal white adipose tissue (eWAT) mass did not differ among groups (Figure 4I). The kidney index was higher in T2D, Metformin, and DF-L than in NC, while DF-H showed a significant reduction versus T2D (*p* < 0.05; Figure 4J).

Serum alanine aminotransferase (ALT) was significantly elevated in T2D (*p* < 0.05; Figure 4K). Compared with T2D, ALT was significantly lower in Metformin (*p* < 0.05) and showed a nonsignificant downward trend in DF-L and DF-H. Serum aspartate aminotransferase (AST) did not differ significantly among groups (Figure 4L). Gross liver images together with H&E and Oil Red O staining indicated marked steatosis in T2D-enlarged, pale, greasy livers; hepatocyte hypertrophy with loosened cytoplasm and abundant vacuoles. Metformin, DF-H, and DF-L improved liver color and morphology, attenuated histopathology, reduced lipid droplets, and largely normalized hepatocyte arrangement; minor residual vacuolar change persisted in DF-L (Figure 4M). Collectively, these findings indicate that high-dose distillers’ grain fiber effectively mitigates hepatic lipid accumulation in diabetic mice.

### 3.5. Gut Microbiota Composition and Differential Taxa in T2D Mice Treated with BDG-DF

**Figure 5:**
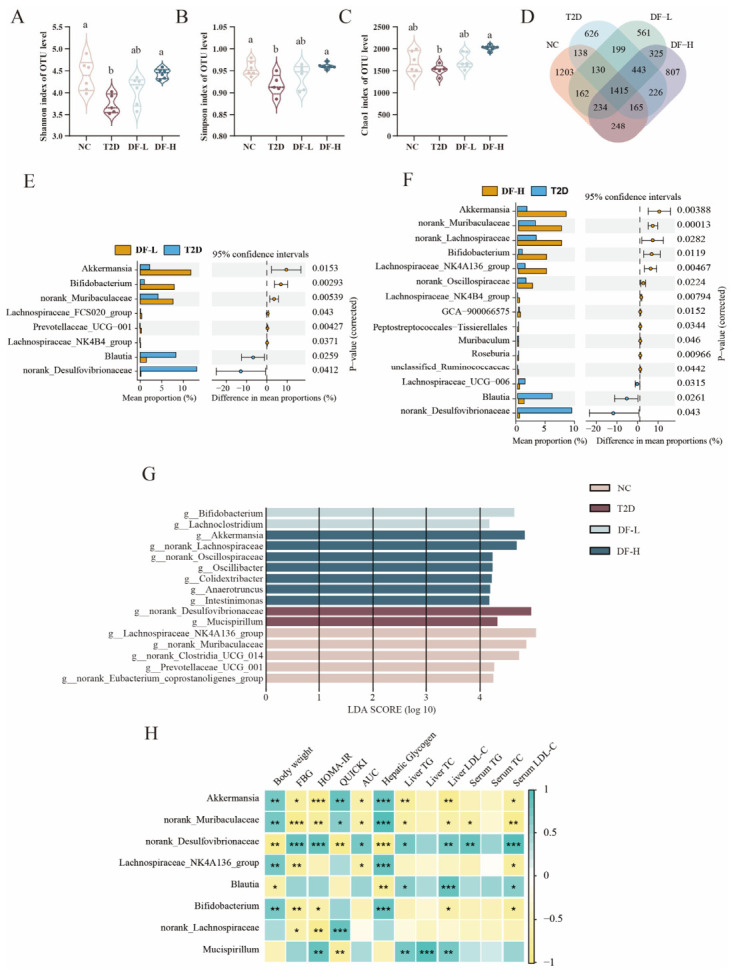
Gut microbiota composition and differential taxa in T2D mice treated with BDG-DF. (**A**) Shannon index. (**B**) Simpson index. (**C**) Chao1 index. (**D**) Venn diagram. STAMP analysis of differential genera between groups: (**E**) DF-L vs. T2D. (**F**) DF-H vs. T2D. (**G**) Histogram of LDA value distribution. (**H**) Correlation analysis between gut microbiota and biochemical indicators. Values are expressed as means ± SD (*n* = 8 for each group). Different letters indicate a significant difference between groups, *p* < 0.05, * *p* < 0.05, ** *p* < 0.01, *** *p* < 0.001.

We profiled the gut microbiota by 16S rRNA sequencing. Alpha-diversity increased with DF-H: Shannon, Simpson, and Chao1 indices were all higher than in T2D (*p* < 0.05; Figure 5A–C), indicating greater richness and evenness. Venn analysis identified 1415 OTUs shared across groups; NC harbored 1203 unique OTUs versus 626 in T2D, while DF-L carried 561 unique OTUs, consistent with fiber-driven remodeling of the T2D microbial pool (Figure 5D). At the phylum level, *Firmicutes* and *Bacteroidetes* dominated (74–89%) without group differences, whereas *Desulfobacterota* was elevated in T2D relative to NC and shifted toward normal with DF-L/DF-H (Figure S1A). At the genus level, major taxa included *Bacteroides*, *norank_Muribaculaceae*, *Lachnospiraceae_NK4A136_group*, *Akkermansia, norank_Lachnospiraceae*, *unclassified_Lachnospiraceae*, and *Bifidobacterium* (Figure S1B). Both DF doses reversed T2D-associated changes in *norank_Muribaculaceae*, *norank_Desulfovibrionaceae*, and *Blautia*, while increasing *Akkermansia* and *Bifidobacterium*.

STAMP detected eight and fifteen genera differing from T2D in DF-L and DF-H, respectively (Figure 5E,F). Across doses, *Akkermansia*, *Bifidobacterium*, and *Lachnospiraceae_NK4B4_group* increased, whereas *norank_Desulfovibrionaceae* and *Blautia* decreased. DF-L additionally enriched *Lachnospiraceae_FCS020_group* and *Prevotellaceae_UCG-001*. DF-H produced broader changes: *norank_Lachnospiraceae, Lachnospiraceae_NK4A136_group*, *norank_Oscillospiraceae*, *GCA-900066575*, *unclassified_Peptostreptococcales-Tissierellales*, *Muribaculum, Roseburia*, and *unclassified_Ruminococcaceae* increased, while *Lachnospiraceae_UCG-006* decreased. LEfSe identified *norank_Desulfovibrionaceae* and *Mucispirillum* as biomarkers of T2D, whereas DF-L was characterized by *Bifidobacterium* and *Lachnoclostridium*, and DF-H by *Akkermansia*, *norank_Lachnospiraceae*, *norank_Oscillospiraceae, Oscillibacter*, *Colidextribacter*, *Anaerotruncus*, and *Intestinimonas* (Figure 5G and Figure S1C).

We next performed an exploratory correlation analysis between representative diabetes-related genera and host metabolic phenotypes (Figure 5H and Figure S1D–K). Several genera altered by BDG-DF intervention, including *Akkermansia*, *Bifidobacterium*, *norank_Muribaculaceae*, and *Lachnospiraceae*-related taxa, showed associations with glycemic, lipid, and hepatic indicators. However, these associations were not uniformly consistent across all phenotypes. Therefore, these microbiota–phenotype relationships should be interpreted as exploratory associations rather than direct evidence of functional mediation. Overall, the correlation results suggest that BDG-DF-induced microbial remodeling is linked to host metabolic changes, but further functional validation is required to determine whether specific taxa directly contribute to the observed metabolic benefits.

### 3.6. Fecal Metabolomic Profiles, SCFAs, and Bile Acids in T2D Mice Treated with BDG-DF

**Figure 6:**
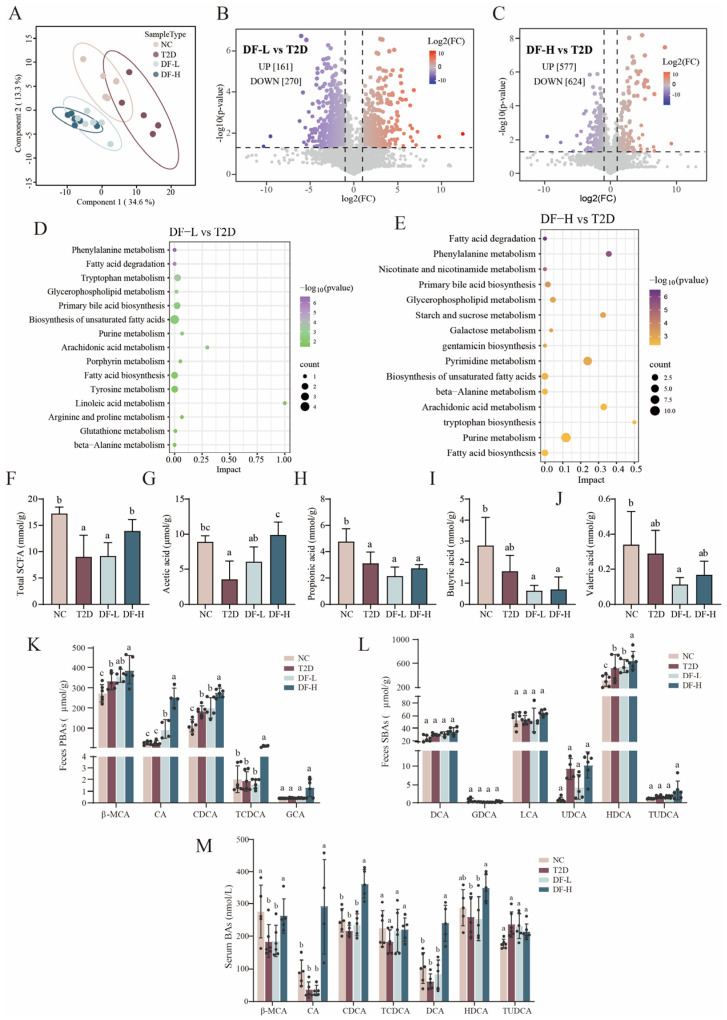
Fecal metabolomic profiles, SCFAs, and bile acids in T2D mice treated with BDG-DF. (**A**) PLS-DA analysis. (**B**) Volcano plots of differential metabolites for DF-L vs. T2D. (**C**) DF-H vs. T2D. TOP15 differential metabolic pathways between the DF-L and T2D group (**D**), DF-H and T2D group (**E**). (**F**–**J**) Effect of dietary fiber from baijiu distillers’ grains on fecal SCFAs in mice. Effect of dietary fiber from baijiu distillers’ grains on fecal bile acids. (**K**) The levels of primary bile acids. (**L**) The levels of secondary bile acids. (**M**) Effect of dietary fiber from baijiu distillers’ grains on serum bile acids. Values are expressed as means ± SD. Different letters indicate a significant difference between groups, *p* < 0.05.

To examine metabolomic sequelae of microbiota remodeling, we conducted untargeted LC-MS/MS profiling, yielding 4643 MS/MS-annotated features across groups. PLS-DA showed clear separation among DF-L, DF-H, and T2D, with a larger displacement under the high dose (Figure 6A). Using |fold-change| > 2 (FC > 2 or <0.5) and *p* < 0.05 versus T2D, volcano plots identified 161 up- and 270 down-regulated metabolites in DF-L (Figure 6B) and 577 up- and 624 down-regulated metabolites in DF-H (Figure 6C). KEGG enrichment implicated broad shifts in amino acid and lipid metabolism, including phenylalanine and β-alanine pathways, fatty acid degradation/biosynthesis, glycerophospholipids, biosynthesis of unsaturated fatty acids, and arachidonic acid metabolism, together with primary bile acid biosynthesis and purine metabolism (Figure 6D,E).

Targeted SCFA quantification by GC-MS showed total fecal SCFAs were reduced in T2D versus NC (*p* < 0.05). DF-H significantly increased total SCFAs toward NC, driven mainly by acetate (*p* < 0.05); DF-L produced a modest acetate rise, while propionate, butyrate, and n-valerate, lower in T2D than NC, were not clearly altered by fiber (Figure 6F–J). Bile acid profiling revealed that in feces, T2D elevated the primary bile acids β-MCA and CDCA and the secondary bile acid HDCA (*p* < 0.05), with limited changes in other species. DF-L had minimal impact, whereas DF-H increased overall fecal bile acids—particularly β-MCA, CA, CDCA, and HDCA (*p* < 0.05; Figure 6K,L). In serum, most bile acids were decreased in T2D relative to NC, with β-MCA showing the largest decline (Figure 6M). DF-H restored serum β-MCA toward NC and significantly raised CA, CDCA, DCA, and HDCA versus T2D. Collectively, these results suggest that high-dose BDG-DF reshaped microbial metabolites and BA profiles in T2D mice, with acetate recovery and BA remodeling representing potential, but not fully resolved, contributors to the observed metabolic improvements.

### 3.7. Bile Acid Metabolism-Related FXR/TGR5 Signaling Markers in T2D Mice Treated with BDG-DF

**Figure 7:**
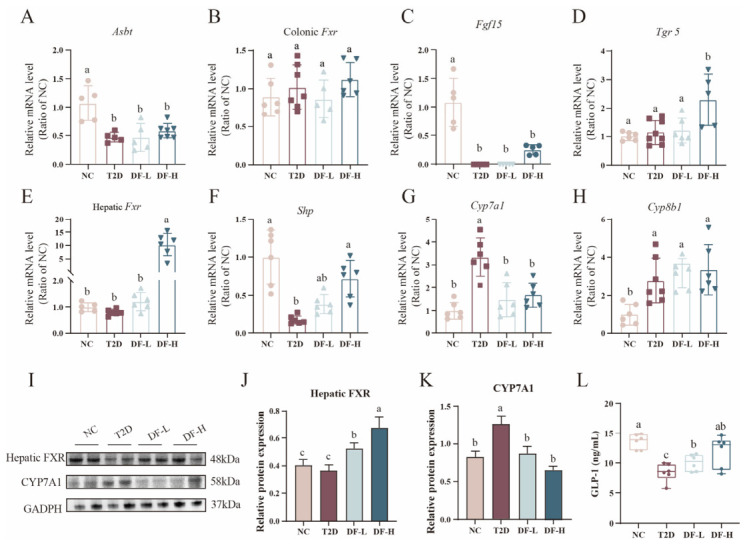
Bile acid metabolism-related FXR/TGR5 signaling markers in T2D mice treated with BDG-DF. Effects of dietary fiber from distillers’ grains on mRNA expression of genes related to bile acid metabolism: (**A**) Colon *Abst*. (**B**) Colon *Fxr*. (**C**) Colon *Fgf15*. (**D**) Colon *Tgr5*. (**E**) Liver *Fxr*. (**F**) Liver *Shp*. (**G**) Liver *Cyp7a1*. (**H**) Liver *Cyp8b1*. (**I**) Western blot analysis of FXR and CYP7A1 in liver tissue: (**J**) relative expression level of FXR protein (**J**), CYP7A1 (**K**). GLP-1 level in serum (**L**). Values are expressed as means ± SD. Different letters indicate a significant difference between groups, *p* < 0.05.

Given the bile acid shifts observed in feces and serum, we profiled the FXR pathway. Intestinal *Asbt* mRNA in DF-H was unchanged versus T2D (Figure 7A), and transcript levels of colonic *Fxr* was unchanged among groups (Figure 7B). *Fgf15*, an FXR-dependent enterohepatic hormone, was scarcely detectable in diabetic colons (Figure 7C). By contrast, *Tgr5* rose with bile acid modulation (Figure 7D), and DF-H significantly increased serum GLP-1 (*p* < 0.05; Figure 7L), consistent with TGR5-driven enteroendocrine activation. In the liver, diabetes upregulated *Cyp7a1* and *Cyp8b1* while downregulating *Shp*. DF-H reversed this pattern: *Cyp7a1* decreased, *Shp* was restored, and hepatic *Fxr* increased markedly (*p* < 0.05; Figure 7E–H). Protein data mirrored the transcripts for FXR and CYP7A1 (Figure 7J,K). Mechanistically, serum enrichment of CA, CDCA, and DCA in DF-H provides ligands that activate hepatic FXR, repress CYP7A1, and curb de novo bile acid synthesis. Reduced bile acid output is expected to diminish intestinal cholesterol absorption and exogenous cholesterol influx, thereby contributing to lower serum TC and LDL-C.

### 3.8. Hepatic Proteomic Profiles in T2D Mice Treated with BDG-DF

**Figure 8:**
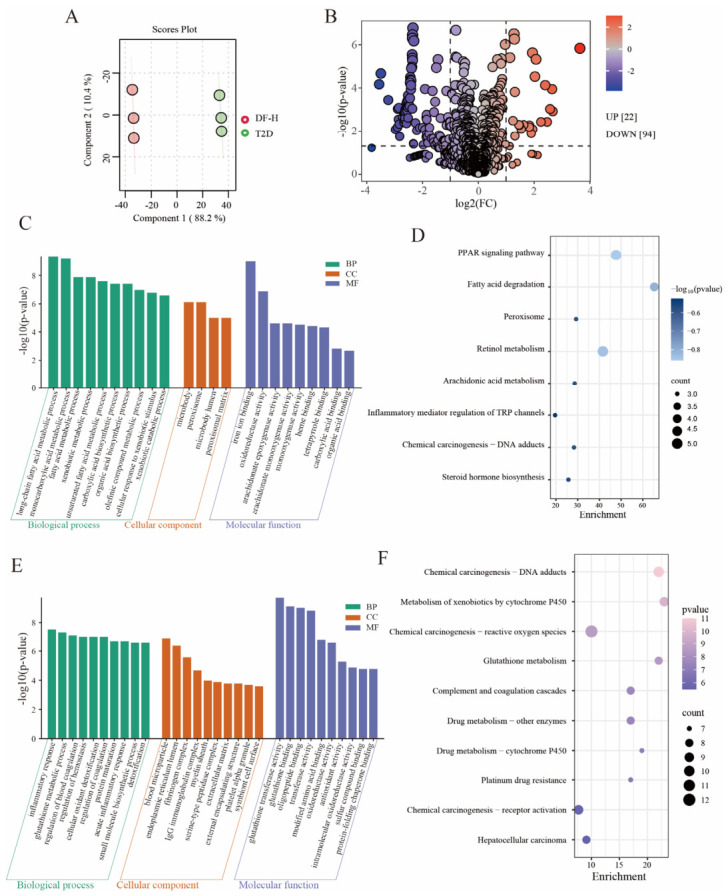
Hepatic proteomic profiles in T2D mice treated with BDG-DF. (**A**) PCA analysis. (**B**) Volcano plot of differentially expressed proteins. GO enrichment analysis results (**C**) and KEGG enrichment analysis (**D**) of up-regulated proteins in DF-H vs. T2D. GO enrichment analysis results (**E**) and KEGG enrichment analysis (**F**) of down-regulated proteins in DF-H vs. T2D.

We next profiled the hepatic proteome to assess T2D-related changes and fiber effects. PCA revealed clear separation between DF-H and T2D with tight within-group clustering, indicating a marked DF-H-driven shift in liver protein expression (*p* < 0.05; Figure 8A). Using |fold change| > 2 (FC > 2 or <0.5) and *p* < 0.05, we identified 116 differentially expressed proteins (DEPs) in DF-H versus T2D-22 up-regulated and 94 down-regulated (Figure 8B). GO enrichment of the 22 up-regulated proteins (top 10 terms; Figure 8C) highlighted biological processes in long-chain fatty acid, monocarboxylate, and general fatty acid metabolism; cellular components centered on microbodies/peroxisomes (including lumen and matrix); and molecular functions involving iron–ion binding, oxidoreductase activity, and arachidonic acid epoxygenase activity.

For the 94 down-regulated proteins (Figure 8E), GO terms were enriched for inflammatory response, glutathione metabolism, and regulation of metabolic processes; cellular components such as blood microparticles, endoplasmic reticulum lumen, and IgG complexes; and molecular functions including glutathione transferase activity, glutathione binding, and oligopeptide binding. KEGG analysis (ranked by −log10 *p*-value) showed up-regulated proteins enriched in PPAR signaling, fatty acid degradation, peroxisome, retinol metabolism, and arachidonic acid metabolism (Figure 8D). Down-regulated proteins were over-represented in chemical carcinogenesis-DNA adducts, xenobiotic metabolism by cytochrome P450, and chemical carcinogenesis-reactive oxygen species (Figure 8F). DIA proteomics comparing T2D and normal livers are provided in Figure S2.

### 3.9. Hepatic Metabolomic Profiles and Proteome–Metabolome Overlap in T2D Mice Treated with BDG-DF

**Figure 9:**
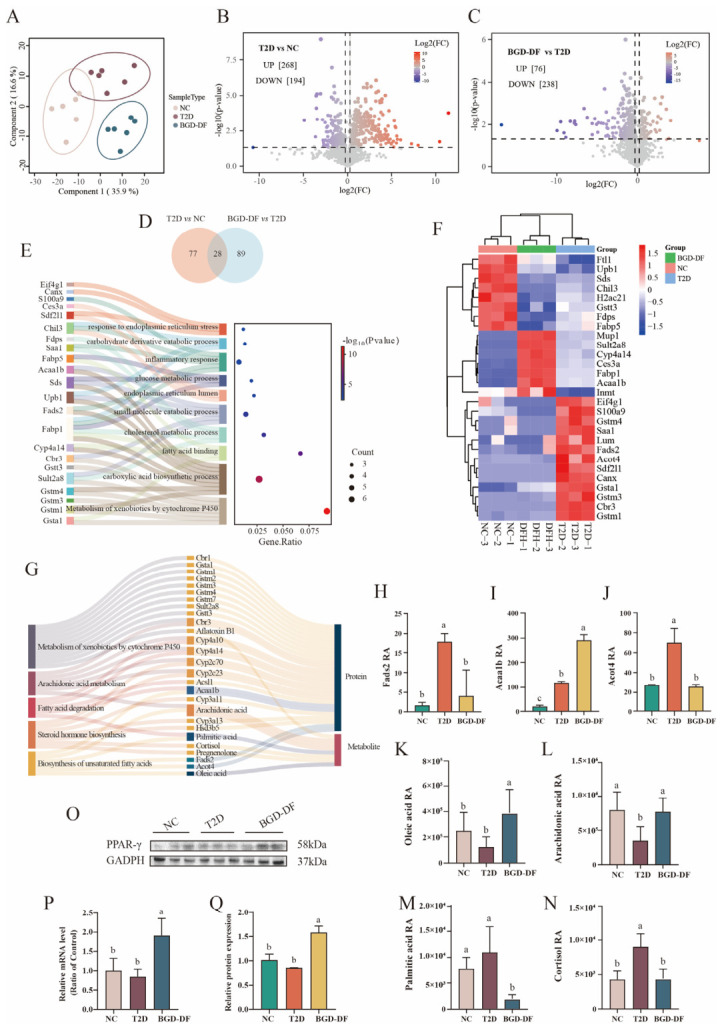
Integrated hepatic proteomic and metabolomic profiles in T2D mice treated with BDG-DF. (**A**) PLS-DA analysis. (**B**) Volcano plots of differential metabolites for T2D vs. NC, (**C**) DF-H vs. T2D. (**D**) Venn diagram. (**E**) Heatmap of relative expression levels of overlapping proteins. (**F**) Sankey diagram of KEGG and GO pathway enrichment. (**G**) Protein and metabolite integrative analysis of the KEGG pathway of Sankey diagram. Relative abundance of key node proteins and metabolites: (**H**) Fads2, (**I**) Acot4, (**J**) Acaa1b1, (**K**) oleic acid, (**L**) arachidonic acid, (**M**) palmitic acid, (**N**) cortisol. (**O**–**Q**) Western blot and mRNA analyses were used to verify the expression of PPAR-γ. Values are expressed as means ± SD. Different letters indicate a significant difference between groups, *p* < 0.05.

In parallel, we profiled the hepatic metabolome to capture T2D-associated shifts. In total, 1477 metabolites with MS/MS matches to reference databases were identified. PLS-DA showed clear separation between DF-H and T2D, indicating a robust fiber-driven remodeling of liver metabolites (Figure 9A). Using |fold change| > 1.2 (FC > 1.2 or <0.83) and *p* < 0.05 versus T2D, volcano plots revealed 268 increases and 194 decreases in T2D relative to NC (Figure 9B), and 76 increases with 238 decreases in DF-H relative to T2D (Figure 9C). KEGG enrichment of differential metabolites highlighted pathway-level effects. Diabetes chiefly affected ascorbate and aldarate metabolism, vitamin B6 metabolism, and purine metabolism (Figure S3A). High-dose fiber modulated vitamin B6 metabolism, steroid hormone biosynthesis, cytochrome P450-mediated xenobiotic metabolism, and purine metabolism in T2D mice (Figure S3B). Lipid-related pathways were also influenced, including biosynthesis of unsaturated fatty acids, fatty acid elongation, fatty acid degradation, and arachidonic acid metabolism. In the proteomic contrasts, 104 DEPs were identified for NC vs. T2D and 116 for DF-H vs. T2D, with 28 proteins overlapping between the two comparisons (Figure 9D). Hierarchical clustering of these 28 proteins showed that the DF-H profile was more similar to NC (Figure 9F). GO/KEGG enrichment of the overlapping set (Figure 9E) highlighted cytochrome P450-mediated xenobiotic metabolism, carbohydrate-derivative catabolic processes, inflammatory response, glucose metabolic processes, cholesterol metabolism, and fatty acid binding.

### 3.10. Integrated Hepatic Proteomic and Metabolomic Pathway Analysis in T2D Mice Treated with BDG-DF

To further elucidate the molecular basis underlying the metabolic effects of BDG-DF, we integrated the hepatic proteomic and metabolomic datasets at the pathway level. KEGG enrichment identified 35 pathways associated with differentially expressed proteins and 12 pathways associated with DF-H-regulated differential metabolites, with five shared pathways: biosynthesis of unsaturated fatty acids, steroid hormone biosynthesis, fatty acid degradation, cytochrome P450-mediated xenobiotic metabolism, and arachidonic acid metabolism. These shared pathways indicate that the hepatic effects of BDG-DF were not restricted to isolated proteins or metabolites, but converged on interconnected metabolic processes related to lipid handling, steroid hormone metabolism, xenobiotic metabolism, and arachidonic acid turnover. Nevertheless, this integrative analysis remains primarily pathway-based and should be interpreted as hypothesis-generating rather than direct functional validation. The pathway map (Figure 9G) shows that xenobiotic metabolism recruited the largest protein cohort—*Cbr1*, *Gsta1*, *Gstm1/2/3/4/7*, *Sult2a8*, *Gstt3*, and *Cbr3* (11 proteins)—mostly belonging to the glutathione S-transferase family and primarily involved in aflatoxin B1 detoxication, with limited direct linkage to lipid metabolism.

By contrast, molecules within the other pathways displayed diabetes-relevant regulatory roles. Furthermore, metabolite analysis revealed that cortisol levels were significantly elevated in T2D liver and restored to NC levels by fiber intervention (*p* < 0.05; Figure 9N), suggesting that lowering hepatic cortisol may contribute to improved insulin resistance. Across lipid-related pathways, DF-H significantly decreased *Fads2* and *Acot4* while increasing Acaa1 relative to T2D (*p* < 0.05; Figure 9H–J). Oleic acid (OA) and arachidonic acid (AA) were reduced in T2D vs. NC but were significantly elevated by DF-H (*p* < 0.05; Figure 9K,L), whereas palmitic acid (PA) was markedly lower in DF-H than in both NC and T2D (*p* < 0.05; Figure 9M). Given that OA, AA, and PA can serve as PPAR agonists, we assessed PPAR signaling and confirmed PPAR-γ activation in DF-H by both Western blot and mRNA analyses (*p* < 0.05; Figure 9O–Q).

## 4. Discussion

Cereal-derived dietary fiber has attracted considerable interest as a dietary strategy for glucose and lipid metabolism regulation. The metabolic effects of dietary fiber are highly structure-dependent, and fermentation can substantially remodel fiber architecture and functionality [17]. Dietary fiber derived from baijiu distillers’ grains (BDG-DF) emerge as a promising yet underexplored resource. During baijiu production, distillers’ grains undergo extensive microbial saccharification and fermentation, providing favorable conditions for natural structural remodeling. Nevertheless, the structural characteristics and metabolic relevance of BDG-DF have remained largely unexplored. In this study, BDG-DF displayed an evenly distributed monosaccharide profile, with substantial proportions of glucose (28.35%), mannose (34.83%), and xylose (35.14%). Compared with many conventional cereal-derived dietary fibers, the proportions of mannose and xylose in BDG-DF were relatively high, suggesting that prolonged fermentation and distillation may have reshaped the composition of the insoluble fiber fraction [6]. This compositional pattern is likely linked to the complex processing history of baijiu production, in which microbial saccharification, fermentation, and subsequent distillation may selectively remove more readily degradable carbohydrates while retaining structurally stable polysaccharide components [18].

From a functional perspective, both mannose- and xylose-containing polysaccharide structures may contribute to the biological activity of BDG-DF. Previous studies have shown that mannose is associated with immune and metabolic regulation, including modulation of gut microbiota composition and alleviation of diet-induced metabolic dysfunction [19]. In diabetic models, mannose supplementation has been reported to promote wound healing, partly by facilitating the transition of macrophages from a pro-inflammatory M1 phenotype to an anti-inflammatory M2 phenotype [20]. In parallel, xylose-rich cereal polysaccharides, particularly those related to arabinoxylan- or xylan-type structures, have been widely implicated in gut microbial fermentation, short-chain fatty acid production, and improvement of host metabolic homeostasis [21]. Therefore, the coexistence of relatively high mannose and xylose contents in BDG-DF may provide a structural basis for its capacity to influence both microbial metabolism and host metabolic signaling. Beyond monosaccharide composition, BDG-DF showed increased structural ordering, enhanced viscoelastic behavior, and a more open surface morphology. Such physical characteristics may influence microbial accessibility and fermentation behavior [22], although direct structure–function validation requires further fine-structure analysis.

Building on the distinctive structural features of BDG-DF, we next employed a T2D mouse model to evaluate its regulatory potential on glucose–lipid homeostasis. In this context, we found that BDG-DF reshaped the gut microbial community toward taxa associated with barrier integrity and metabolic benefits. DF-H increased α-diversity and enriched *Akkermansia*, a genus that has been inversely associated with T2D and reported to protect against high-glucose-induced β-cell injury in previous studies [23]. *Bifidobacterium* was restored by DF-L/DF-H (*p* < 0.05), consistent with lowered glycemia and improved insulin/adipokine profiles in similar models [24]. In contrast, *Blautia* was elevated in T2D but decreased after fiber, aligning with cohort data linking *Blautia* to T2D and with declines observed under Portulaca extract [25]. DF-H also partly rescued the *Lachnospiraceae* NK4A136 group and increased norank_*Lachnospiraceae* (*p* < 0.05), taxa reported to rebound with therapy and alleviate insulin resistance [26]. *Mucispirillum* rose in T2D but normalized under DF-H and has been positively correlated with metabolic risk markers in diabetic rodents [27]. Finally, norank_*Desulfovibrionaceae*—a sulfate-reducing H_2_S/LPS-producing lineage—was elevated in T2D and reduced only by DF-H [28], whereas *norank_Muribaculaceae* declined in T2D and recovered under DF-L/DF-H, more strongly with DF-H, in line with reports of IRS/PI3K/Akt activation and improved glucose uptake/glycogenesis [29]. Together, the enrichment of *Akkermansia*, *Bifidobacterium, Muribaculaceae,* and selected *Lachnospiraceae* suggests that BDG-DF reshaped the gut microbial community in parallel with SCFA production, bile acid remodeling, and improved glucose–lipid homeostasis. Nevertheless, these microbiota-related findings remain primarily correlative, and further functional validation is required to determine whether these taxa directly mediate the metabolic benefits of BDG-DF.

Untargeted LC-MS/MS indicated that both low- and high-dose BDG-DF engaged fatty acid (FA) degradation pathways, countering T2D signatures tightly intertwined with insulin resistance, where diminished insulin sensitivity elevates adipose lipolysis and circulating free FAs, disrupts insulin signaling, and amplifies hepatic gluconeogenesis [30]. GC-MS profiling showed that DF-H significantly increased total SCFAs, mainly driven by acetate, whereas propionate, butyrate, and n-valerate, which were lower in T2D mice, were not clearly restored. Branched SCFAs were largely unchanged, with modest decreases in butyrate/isovalerate under DF-L. The acetate increase was accompanied by the expansion of putative acetate-producing taxa, including *Bifidobacterium, Akkermansia*, and *norank_Muribaculaceae.* Although SCFAs have been reported to regulate host metabolism through receptors such as GPR41/GPR43 and downstream AMPK-related pathways [31], these signaling events were not directly examined in the present study and therefore should be considered as potential mechanisms requiring further validation. Differential-pathway analysis also indicated that BDG-DF affected primary bile acid biosynthesis, consistent with microbiota–bile acid crosstalk that may modulate FXR/TGR5-related signaling and FA-catabolic programs in metabolic tissues [32].

Bile acids (BAs) circulate along the enterohepatic axis, where they are synthesized from hepatic cholesterol, secreted into the intestine to facilitate lipid absorption, and largely reabsorbed for portal return to the liver [33]. In the present study, DF-H was associated with increases in several fecal and serum BA species. These changes also suggest a shift in BA pool composition, indicating that BDG-DF may be associated with remodeling of BA homeostasis rather than simply increasing total BA levels. This compositional shift is physiologically important because different BAs differ markedly in microbial origin, receptor activity, hydrophobicity, and host metabolic effects [34]. In feces, DF-H increased the primary BAs β-MCA, CA, and CDCA, together with the secondary BA HDCA. This pattern suggests that BDG-DF may have enhanced intestinal BA turnover and microbial transformation while avoiding indiscriminate accumulation of strongly hydrophobic and potentially cytotoxic secondary BAs. Notably, β-MCA is an endogenous intestinal FXR antagonist, and its elevation may attenuate intestinal farnesoid X receptor (FXR) signaling, which has been associated with improved insulin sensitivity and reduced diet-induced metabolic dysfunction [35]. HDCA likewise antagonizes intestinal FXR. By relieving FXR tone, it can favor a CYP8B1-centered route that accelerates cholesterol conversion to BAs and reduces hepatic TG and cholesterol accumulation, mitigating NAFLD-like sequelae [36]. In contrast, CA and CDCA are agonists for FXR and TGR5, the latter promoting GLP-1 release to enhance insulin sensitivity and lower glycemia [37]. Given these opposing ligands, the net intestinal FXR state required experimental resolution.

BAs are not only detergents involved in lipid absorption but also signaling molecules that regulate host glucose and lipid metabolism through receptors such as FXR and TGR5 [38]. Importantly, different BA species exhibit distinct, and sometimes opposing, receptor activities [39]. Therefore, the metabolic consequence of BDG-DF-induced BA remodeling should be interpreted based on the composition and tissue distribution of the BA pool rather than total BA abundance alone. In the present study, DF-H increased fecal β-MCA, CA, CDCA, and HDCA, and elevated serum CA, CDCA, DCA, and HDCA. This pattern suggests that BDG-DF reshaped enterohepatic BA signaling in a compartment-specific manner. In the intestine, β-MCA and HDCA have been reported to act as FXR antagonists, whereas CA and CDCA can function as FXR agonists and also contribute to TGR5 activation [40]. Thus, the simultaneous increase in these BA species may generate a balanced or even offsetting effect on intestinal FXR signaling [41]. This interpretation is consistent with our observation that intestinal Asbt expression was not increased, colonic Fxr expression remained unchanged, and Fgf15 was barely detectable in diabetic colons. These results suggest that BDG-DF did not predominantly improve host metabolism through activation of the intestinal FXR–FGF15 axis. Instead, the increased Tgr5 expression and elevated serum GLP-1 level indicate that the intestinal BA response was more closely associated with activation of the TGR5–GLP-1 pathway [42]. This pathway may contribute to improved glucose homeostasis by enhancing glucose-stimulated insulin secretion, reducing glucagon release, delaying gastric emptying, and lowering postprandial glycemia [43].

By contrast, in the liver, the serum enrichment of CA, CDCA, and DCA provides a plausible ligand basis for hepatic FXR activation. Consistent with this, DF-H markedly increased hepatic Fxr and Shp expression and reduced Cyp7a1 expression, with Western blot results confirming increased FXR protein and decreased CYP7A1 protein. Mechanistically, hepatic FXR activation induces SHP, which represses CYP7A1 transcription by inhibiting LRH-1- and HNF4-dependent promoter activation. Therefore, the DF-H-induced decrease in CYP7A1 suggests enhanced hepatic FXR–SHP negative feedback on de novo BA synthesis. Taken together, these findings suggest that BDG-DF was associated with changes in BA-related signaling in a tissue-specific manner. In the intestine, FXR signaling was not clearly activated. This may be because FXR-antagonistic BAs counterbalanced the effects of FXR agonists. In contrast, intestinal TGR5–GLP-1-related signaling appeared to be enhanced. In the liver, BDG-DF was associated with increased FXR–SHP signaling and reduced CYP7A1 expression. These changes suggest that BDG-DF-associated BA remodeling may contribute to improved glycemic control, reduced serum TC and LDL-C levels, and alleviation of hepatic steatosis.

Integrated liver proteome–metabolome analysis identified steroidogenesis and lipid pathways as key effectors of BDG-DF action. Hepatic cortisol—a key effector in the steroid hormone biosynthesis pathway—was significantly elevated in T2D mice (*p* < 0.05). This finding is consistent with reports that 24% of treatment-refractory T2D patients exhibit hypercortisolism, frequently accompanied by hypertension and obesity, and with evidence that sustained cortisol excess impairs insulin signaling and reduces glucose uptake in muscle and adipose tissue, thereby promoting insulin resistance [44]. Notably, DF-H was associated with a reduction in hepatic cortisol levels. The cortisol level in the DF-H group was comparable to that in the normoglycemic control group. This result suggests that BDG-DF may help reduce hepatic cortisol accumulation. It may also be associated with improved insulin resistance in T2D mice. At the gene-regulatory level, DF-H also remodeled hepatic lipid metabolism. Fads2 encodes Δ6-desaturase, which participates in the biosynthesis of long-chain polyunsaturated fatty acids, while Acot4 is involved in acyl-CoA hydrolysis and dicarboxylate metabolism [45]. Compared with the T2D group, DF-H significantly downregulated Fads2 and Acot4 while upregulating Acaa1. Given that Acaa1 participates in fatty acid β-oxidation-related metabolism, its upregulation in the DF-H group suggests a potential shift toward improved hepatic fatty acid handling. However, because fatty acid oxidation flux and de novo lipogenesis were not directly measured in this study, these proteomic changes should be interpreted as pathway-level evidence rather than direct proof of enhanced β-oxidation or suppressed lipogenesis. Consistent with these protein-level changes, DF-H reshaped the hepatic fatty acid profile: oleic acid (OA) and arachidonic acid (AA) were increased relative to T2D and restored toward normal, whereas palmitic acid (PA) was markedly reduced. OA, AA, and PA can act as endogenous ligands or modulators of PPAR-related signaling [46]. Therefore, the recovery of OA and AA, together with reduced PA and enrichment of PPAR-related pathways, supports the possibility that BDG-DF modulated hepatic lipid-metabolic signaling. Nevertheless, whether these changes directly enhance lipid oxidation or suppress hepatic lipogenesis requires further functional validation.

Although the integrated proteomic and metabolomic analysis provided a coherent view of BDG-DF-associated hepatic metabolic remodeling, this approach remains primarily descriptive. The convergence of differential proteins and metabolites on unsaturated fatty acid biosynthesis, fatty acid degradation, steroid hormone biosynthesis, cytochrome P450-mediated xenobiotic metabolism, and arachidonic acid metabolism suggests that these pathways may participate in the metabolic effects of BDG-DF. In particular, coordinated changes in Fads2, Acot4, Acaa1, OA, AA, PA, and cortisol support a potential link between BDG-DF intervention and hepatic lipid-metabolic reprogramming. Nevertheless, pathway enrichment and correlation-based integration cannot determine whether these proteins or metabolites are causal mediators. Therefore, these multi-omics findings should be considered as a network-level framework for hypothesis generation. Future studies should further validate the key proteins and metabolites identified in this study. Enzyme activity assays are needed to examine lipid-metabolic enzymes. Isotope-tracing experiments should also be performed to evaluate fatty acid flux. In addition, pharmacological or genetic approaches targeting PPAR-γ and related pathways are required to confirm their functional roles.

However, several limitations related to structural characterization, mechanistic validation, and translational application should be acknowledged. First, the HFD/STZ model used in this study includes both HFD-induced insulin resistance and STZ-induced pancreatic β-cell injury. Because STZ has direct pancreatotoxic effects, this model may include a substantial β-cell injury component and should not be interpreted as a purely diet-induced insulin resistance model. Future studies using additional diabetic animal models, longer-term dietary intervention, dose–response evaluation, and direct assessment of pancreatic β-cell function, insulin signaling, and hepatic lipid metabolism are needed to verify the reproducibility and generalizability of the metabolic effects of BDG-DF. Second, the present structural characterization mainly described the overall compositional and physicochemical features of the insoluble BDG-DF fraction, including monosaccharide composition, FT-IR spectra, X-ray diffraction, rheological behavior, particle size distribution, and surface morphology. Advanced fine-structure analyses, such as molecular weight distribution, NMR spectroscopy, and glycosidic linkage analysis, were not performed. In addition, typical dietary fiber functional properties, including water-holding capacity, oil-holding capacity, swelling capacity, glucose adsorption, cholesterol adsorption, and bile-acid-binding capacity, should be evaluated in future studies. The present results suggest that BDG-DF intervention was associated with microbial remodeling, SCFA production, changes in bile acid profiles, and hepatic metabolic signaling. However, several key mechanisms were not directly examined in this study. These include intestinal tight junction proteins, circulating or fecal LPS levels, GPR41/GPR43 and AMPK signaling, and microbial functional changes based on metagenomics or metatranscriptomics. Therefore, future studies should combine fine-structure analysis, dietary fiber functional assays, intestinal barrier evaluation, endotoxin quantification, SCFA receptor/AMPK signaling validation, and microbial functional omics to establish a more causal structure–microbiota–host metabolic mechanism.

From a translational perspective, BDG-DF may have potential value as a functional dietary fiber ingredient because BDG is an abundant by-product of the baijiu industry, and its valorization may contribute to both functional food development and by-product utilization. Nevertheless, several technological issues must be addressed before industrial application. The composition of BDG-DF may vary depending on raw materials, fermentation starters, fermentation duration, distillation process, and post-treatment conditions; therefore, standardized extraction procedures and quality control indicators, including dietary fiber composition, monosaccharide profile, particle size, hydration properties, and residual chemical levels, are necessary to ensure batch-to-batch consistency. Moreover, the present extraction procedure was conducted at the laboratory scale, and its scalability, production yield, cost-effectiveness, environmental impact, and compatibility with food-grade processing require further optimization. The technological stability of BDG-DF during food processing and storage, including heat treatment, pH variation, drying, and incorporation into complex food matrices, also remains to be evaluated. Potential applications may include dietary-fiber-enriched cereal foods, meal-replacement products, functional beverages, fermented foods, or metabolic health-oriented nutritional formulations. Further studies should assess sensory properties, processing stability, long-term safety, and efficacy in human populations before BDG-DF can be developed as a practical functional food ingredient.

## 5. Conclusions

**Figure 10:**
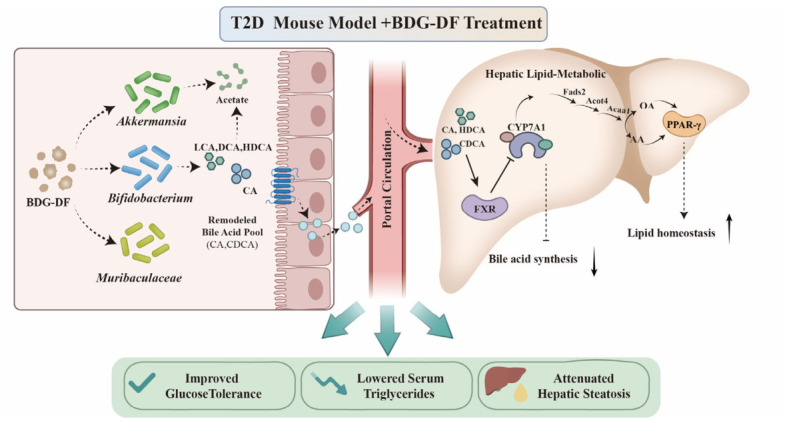
Schematic overview of the association between BDG-DF intervention, bile acid homeostasis, and hepatic lipid metabolism.

In conclusion, BDG-DF improved glucose–lipid homeostasis, alleviated dyslipidemia, and reduced hepatic steatosis in a T2D mouse model. These effects were associated with gut microbiota remodeling, an acetate-dominant SCFA response, bile acid pool remodeling, intestinal TGR5-GLP-1-related changes, hepatic FXR-CYP7A1 feedback regulation, and PPAR-γ-associated hepatic lipid-metabolic pathways (Figure 10). However, these findings remain mainly association-based and pathway-level evidence. Several limitations should be noted. This study was performed in a single animal model, and its relevance to humans requires further validation. In addition, the causal roles of specific gut bacteria, SCFAs, bile acids, FXR/TGR5 signaling, PPAR-γ regulation, β-oxidation, and hepatic lipogenesis were not directly confirmed. Future studies using fecal microbiota transplantation, receptor-based assays, isotope tracing, enzyme activity assays, and targeted functional validation are needed. From a translational perspective, BDG-DF may serve as an upcycled functional dietary fiber ingredient from baijiu industrial by-products for metabolic health-oriented food development. Further work should evaluate scalable food-grade extraction, batch stability, processing and storage stability, safety, sensory compatibility, and efficacy in human populations.

## Data Availability

The original contributions presented in this study are included in the article/Supplementary Materials. Further inquiries can be directed to the corresponding authors.

## Supplementary Materials

The following supporting information can be downloaded at: https://www.mdpi.com/article/10.3390/foods15122163/s1, Figure S1: Effects of BDG-DF on gut microbiota composition and representative differential genera in T2D mice; Figure S2: Hepatic proteomic changes between T2D and NC mice; Figure S3: KEGG pathway enrichment analysis of hepatic differential metabolites; Figure S4: Original western blot of Hepatic FXR, CYP7A1, and PPAR-γ; Table S1: The composition of insoluble dietary fiber in BDG-DF; Table S2: Primers of target genes.
