# Supplementary material for: Dietary Fiber from Baijiu Distillers’ Grains Improves Glucose–Lipid Homeostasis via Gut–Liver Metabolic Remodeling
Source: Foods. 2026 Jun 15;15(12):2163. doi: 10.3390/foods15122163 (PMC13298373; doi:10.3390/foods15122163)
Supplement: Supplementary file 1 [file foods-15-02163-s001.zip › foods-4320130-supplementary.pdf]

Supplementary data

Section 1. Methods

1.1. Monosaccharide composition analysis.

The monosaccharide composition of BDG-DF was determined by HPLC following acid hydrolysis and PMP derivatization. Briefly, mixed standards of mannose acid, mannose, Ribose, glucose acid, galacturonic acid, glucose, galactose, xylose, arabinose, and fucose were prepared to construct calibration curves. Approximately 5 mg of BDG-DF was hydrolyzed with 5 mL of 2 mmol L^{-1} trifluoroacetic acid at 121 °C for 2 h. After cooling to room temperature, methanol was added and the solvent was removed under a gentle stream of nitrogen. The residue was re-dissolved in ultrapure water and derivatized with 0.4 mol L^{-1} PMP in methanol in the presence of 0.6 mol L^{-1} NaOH at 70 °C for 1 h. The reaction was then neutralized with 0.3 mol L^{-1} HCl, and the derivatized monosaccharides were extracted three times with chloroform to remove excess PMP. The aqueous phase was filtered through a 0.45 μm membrane prior to analysis. HPLC analysis was performed using a Shimadzu LC-20AD system equipped with a UV detector and an Xtimate C18 ((Shimadzu, Japan) column maintained at 30 °C. The mobile phase consisted of 0.05 mol L^{-1} KH_2PO_4 buffer (pH 6.7) and acetonitrile (83:17, v/v), with a flow rate of 1.0 mL min^{-1}. Detection was conducted at 250 nm with an injection volume of 20 μL.

1.2. Particle size distribution analysis.

Particle size distribution of BGD-DF was determined using a laser diffraction particle size analyzer (Bettersize 2600, Bettersize Instruments, China). The real and imaginary parts of the refractive index were set to 1.45 and 1.33, respectively. The fiber suspension was added dropwise into the recirculating dispersion unit under continuous stirring until the obscuration reached 8–13%. Measurements were conducted at a pump speed of 30% with a run time of 90 s per measurement, and the stirring speed was maintained at 1600 rpm. After reaching the target obscuration, the suspension was ultrasonicated for 30 s to ensure adequate dispersion, and the particle size distribution was recorded. The characteristic diameters D10, D50, and D90 were reported as the particle sizes corresponding to 10%, 50%, and 90% of the cumulative volume distribution, respectively.

1.3. Fourier transform infrared spectroscopy (FT-IR) analysis.

FT-IR spectra of the BDG-derived dietary fiber (BDG-DF) samples were recorded using a Fourier transform infrared spectrometer in the wavenumber range of 4000–400 cm^{-1}. Briefly, the polysaccharides were thoroughly mixed with spectral-grade KBr, finely ground, and compressed into translucent pellets using a tablet press prior to measurement.

1.4. X-ray diffraction (XRD) analysis.

The relative crystallinity of the samples was determined using an X-ray

diffractometer (SMARTLAB 3 kW, Rigaku, Japan). Measurements were performed in the overlapping scan mode at an operating voltage of 36 kV and a current of 20 mA. Diffraction patterns were collected over a 2θ range of 5°–60° at a scanning rate of 2°/min.

1.5. Scanning electron microscopy (SEM) analysis.

A small amount of BGD-DF was mounted onto the specimen stub using a conductive adhesive, and loosely attached particles were gently removed with a nitrogen stream. The sample was then sputter-coated with a thin layer of gold and again lightly blown with nitrogen to remove any residual loose material. Subsequently, the surface morphology of BGD-DF was examined using a scanning electron microscope (Thermo Fisher Scientific, Eindhoven, The Netherlands) equipped with a CeB6_66 filament electron gun.

1.6. Dynamic rheological measurements.

BGD-DF was dispersed in deionized water and thoroughly mixed, and the pH was adjusted to 6.5. The dispersion was then diluted to obtain a 2% (w/w) suspension, followed by homogenization at 10,000 rpm for 1 min. The suspension was stored at 4 °C overnight to allow complete swelling. Prior to analysis, samples were equilibrated at room temperature for 2 h. Dynamic rheological properties were measured using a hybrid rheometer (Discovery HR20, TA Instruments, USA). All oscillatory measurements were performed within the linear viscoelastic region, and an oscillatory strain of 0.1% was applied for the dynamic rheological tests of BGD-DF

Table S1 The composition of insoluble dietary fiber in baijiu distiller's grains.

Table S2 Primers of target genes.

Fig. S1 Effects of BDG-DF on gut microbiota composition and representative differential genera in T2D mice. (A) Relative abundance at phylum level. (B) Relative abundance at gene level. (C) LEfSe analysis of biomarkers in each group. Effects of dietary fiber from baijiu distiller's grains on gut microbiota: (A) *Akkermansia,* (B) *Bifidobacterium,* (C) *Blautia,* (D) *Lachnospiraceae_NK4A136_group,* (E) *Mucispirillum,* (F) *norank_Desulfovibrionaceae,* (G) *norank_Lachnospiraceae,* (H) *norank_Muribaculaceae*.

Fig. S2 Hepatic proteomic changes between T2D and NC mice. (A) PCA analysis (B) Volcano plot of differentially expressed proteins. GO enrichment analysis results (C) and KEGG enrichment analysis (D) of up-regulated proteins in T2D vs. NC. GO enrichment analysis results (E) and KEGG enrichment analysis (F) of down-regulated proteins in T2D vs. NC.

Fig. S3 KEGG pathway enrichment analysis of hepatic differential metabolites. (A) Results of KEGG pathway analysis of differential metabolites between T2D vs. NC. (B) Results of KEGG pathway analysis of differential metabolites between DF-Hvs.T2D.

Figure S4 Original western blot of Hepatic FXR, CYP7A1, and PPAR-γ.

Table S1 The composition of insoluble dietary fiber in baijiu distiller's grains.

composition	concentration（%）
Moisture	3.96 ± 0.08
Protein	2.40 ± 0.35
Lipid	0.34 ± 0.01
Ash	17.83 ± 0.57
Cellulose	57.07 ± 1.03
Hemicellulose	12.26 ± 0.72
Lignin	6.14 ± 0.29

Values are expressed as mean ± SD, n = 3.

Table S2 Primers of target genes.

Gene name	Forward (5'-3')	Reverse (5'-3')
Fxr	CCCCTGCTTGATGTGCTAC	CGTGGTGATGGTTGAATGTC
Cyp7a1	CTGGGCTGTGCTCTGAAGT	GGGAGTTTGTGATGAAGTGGA
Cyp8b1	ACAGCGTGATGGAGGAGAGT	AGGGGAAGAGAGCCACCTTA
Shp	AAGGGCACGATCCTCTTCAA	CTGTTGCAGGTGTGCGATGT
Fgf15	GAAGACGATTGCCATCAAGGA	CGAATCAGCCCGTATATCTTGC
Asbt	TGATGTTTTCTATGGGGTGCAAT	TGAGAGGCATGATTCCAAACTG
Tgr5	GCTCCTGTCAGTCTTGGCCTAT	TTCCTCGAAGCACTCGTAGACA
Gapdh	TGAGGTCAATGAAGGGGTCGT	CCTCGTCCCGTAGACAAAATG
Ppar-γ	ATTGAGTGCCGAGTCTGTGG	ACCTGATGGCATTGTGAGACA

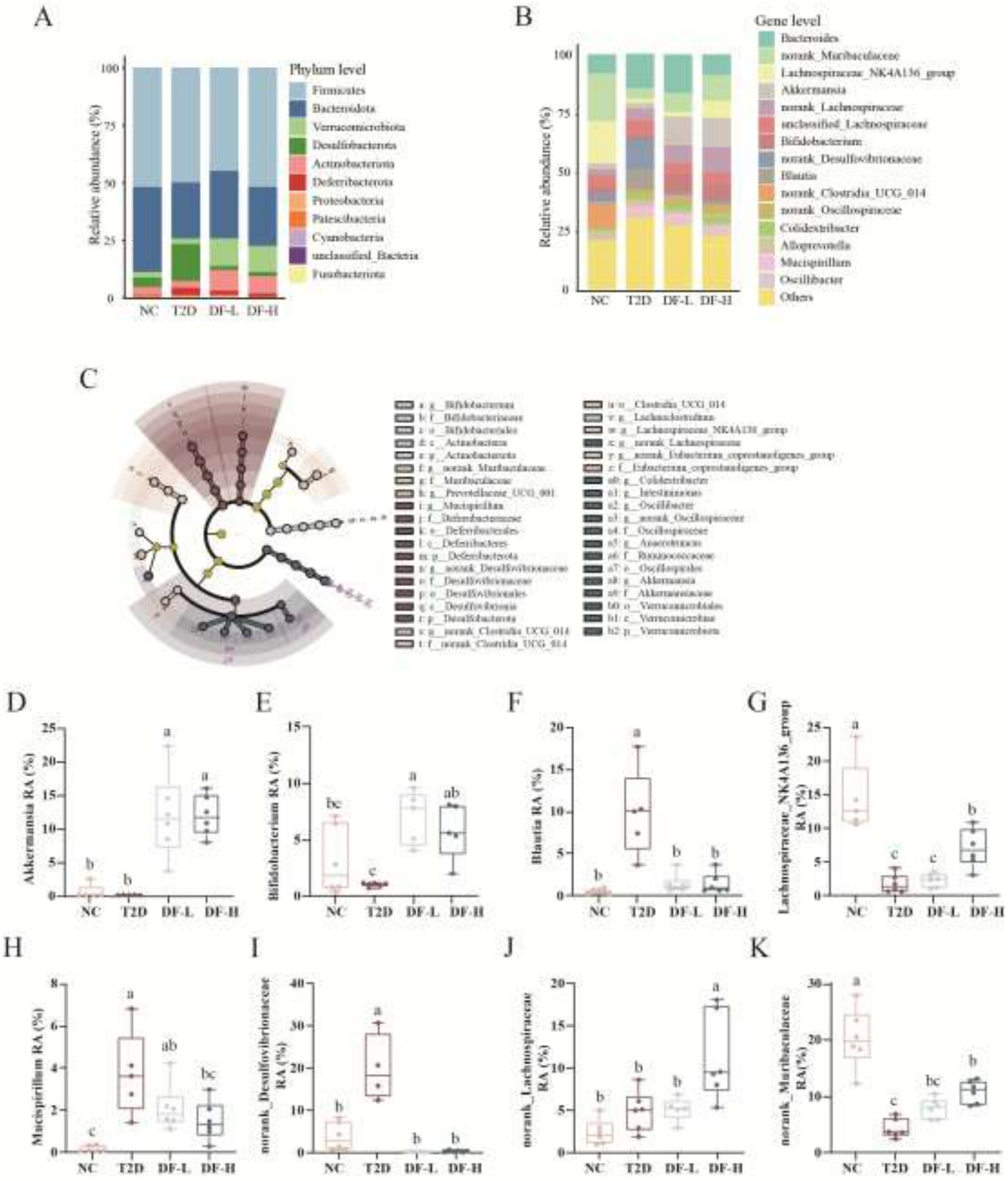

Fig. S1 Effects of BDG-DF on gut microbiota composition and representative differential genera in T2D mice. (A) Relative abundance at phylum level. (B) Relative abundance at gene level. (C) LEfSe analysis of biomarkers in each group. Effects of dietary fiber from baijiu distiller's grains on gut microbiota: (A) *Akkermansia,* (B) *Bifidobacterium,* (C) *Blautia,* (D) *Lachnospiraceae_NK4A136_group,* (E) *Mucispirillum,* (F) *norank_Desulfovibrionaceae,* (G) *norank_Lachnospiraceae,* (H) *norank_Muribaculaceae*.

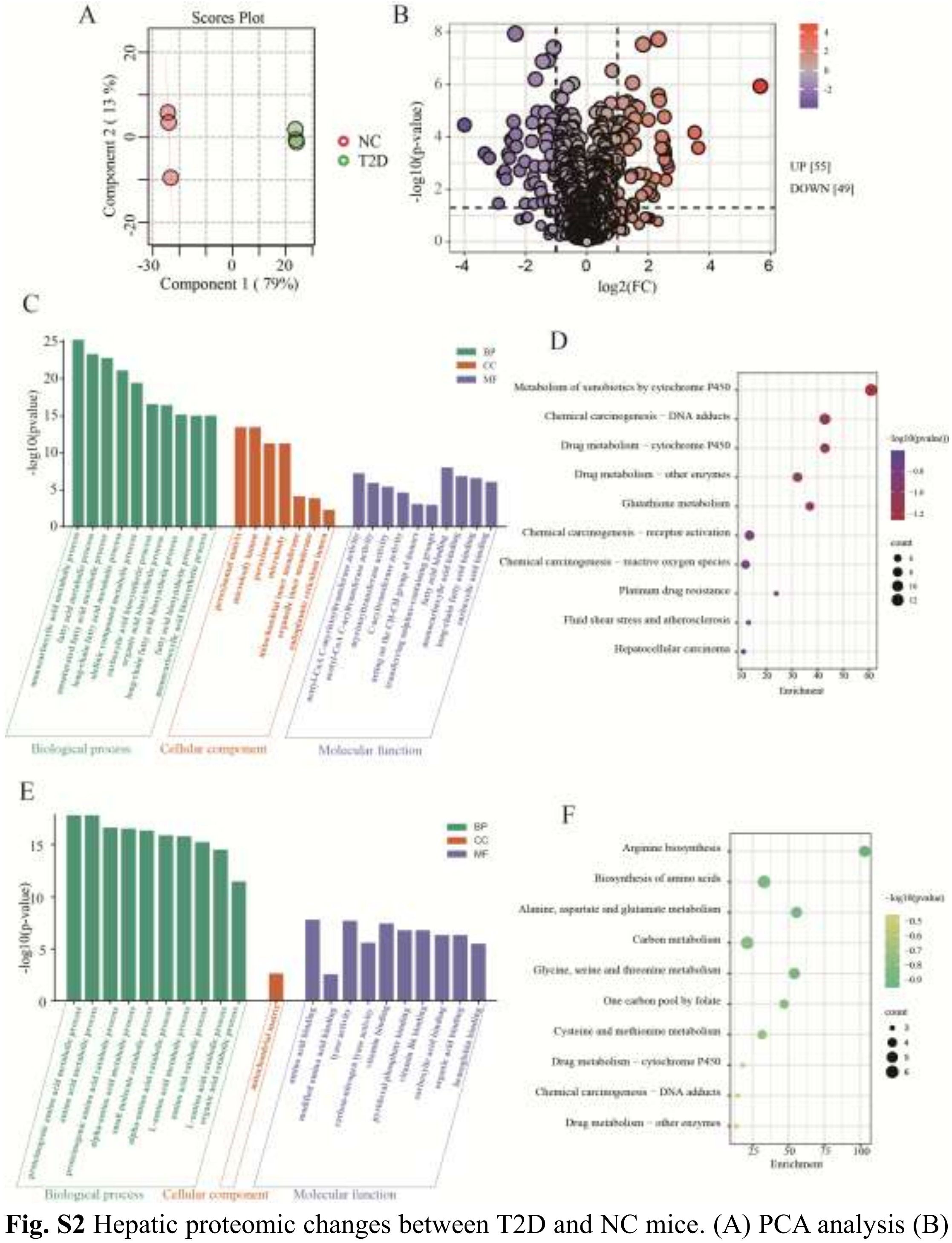

Fig. S2 Hepatic proteomic changes between T2D and NC mice. (A) PCA analysis (B) Volcano plot of differentially expressed proteins. GO enrichment analysis results (C) and KEGG enrichment analysis (D) of up-regulated proteins in T2D vs. NC. GO enrichment analysis results (E) and KEGG enrichment analysis (F) of down-regulated proteins in T2D vs. NC.

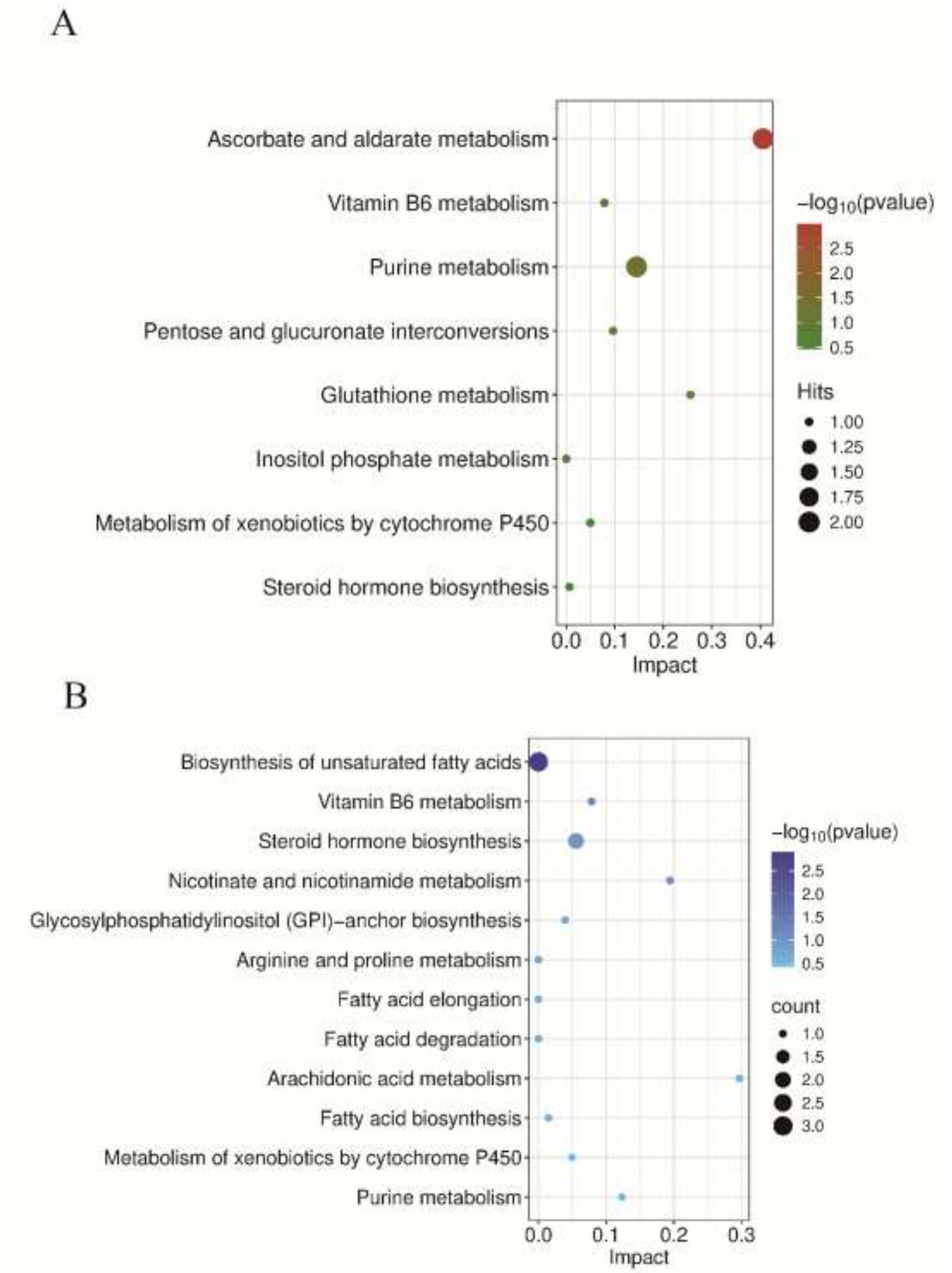

Fig. S3 KEGG pathway enrichment analysis of hepatic differential metabolites. (A) Results of KEGG pathway analysis of differential metabolites between T2D vs. NC. (B) Results of KEGG pathway analysis of differential metabolites between DF-Hvs.T2D.

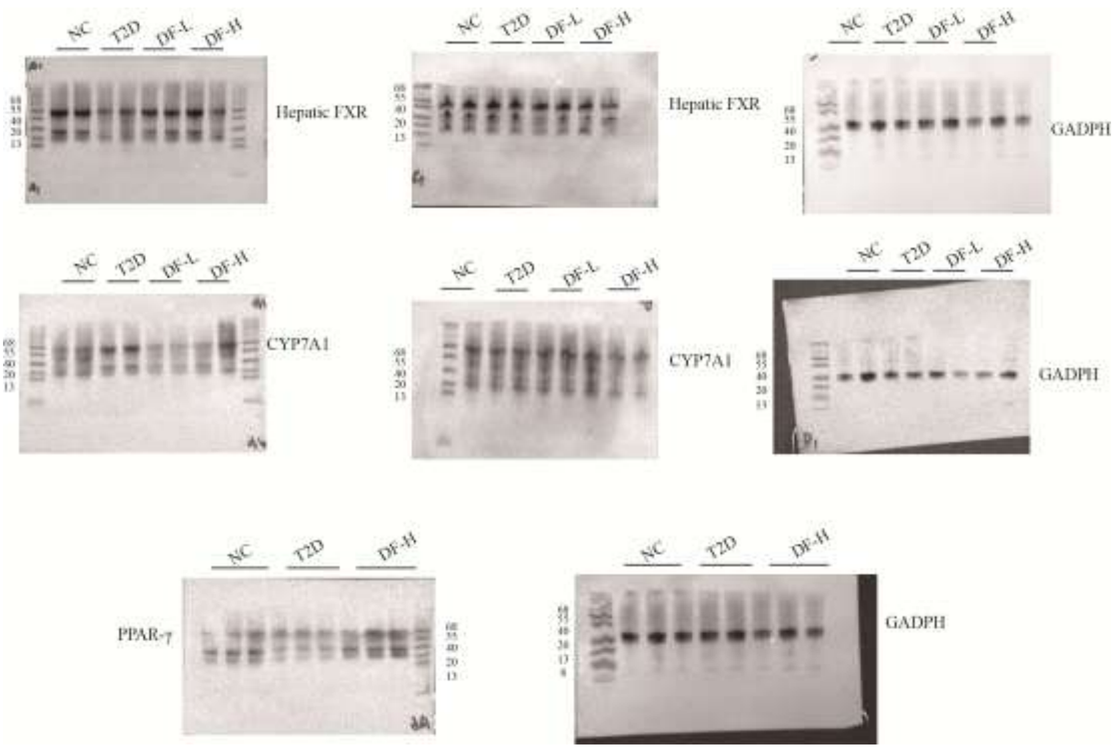

Figure S4 Original western blot of Hepatic FXR, CYP7A1, and PPAR-γ.
